# Transmission of gram-negative antibiotic-resistant bacteria following differing exposure to antibiotic-resistance reservoirs in a rural community: a modelling study for bloodstream infections

**DOI:** 10.1038/s41598-022-17598-x

**Published:** 2022-08-05

**Authors:** Kasim Allel, Lara Goscé, Rafael Araos, Daniel Toro, Catterina Ferreccio, Jose M. Munita, Eduardo A. Undurraga, Jasmina Panovska-Griffiths

**Affiliations:** 1 Institute for Global Health, University College London, 30 Guildford Street, London, WC1N 1EH UK; 2 Department of Disease Control, Faculty of Infectious and Tropical Diseases, London School of Hygiene and Tropical Medicine, London, UK; 3 Antimicrobial Resistance Centre, London School of Hygiene and Tropical Medicine, London, UK; 4 Millennium Initiative for Collaborative Research on Bacterial Resistance (MICROB-R), Santiago, Chile; 5 Instituto de Ciencias e Innovacion en Medicina (ICIM), Facultad de Medicina Clinica Alemana, Universidad del Desarrollo, Santiago, Chile; 6 Advanced Center for Chronic Diseases, Pontificia Universidad Catolica de Chile, Santiago, Chile; 7 Escuela de Gobierno, Pontificia Universidad Católica de Chile, Santiago, RM Chile; 8 Facultad de Medicina, School of Medicine, Pontificia Universidad Católica de Chile, Santiago, Chile; 9 Research Center for Integrated Disaster Risk Management (CIGIDEN), Santiago, RM Chile; 10 CIFAR Azrieli Global Scholars Program, CIFAR, Toronto, Canada; 11 The Big Data Institute, Nuffield Department of Medicine and The Queen’s College, University of Oxford, Oxford, UK

**Keywords:** Risk factors, Bacterial infection, Epidemiology

## Abstract

Exposure to community reservoirs of gram-negative antibiotic-resistant bacteria (GN-ARB) genes poses substantial health risks to individuals, complicating potential infections. Transmission networks and population dynamics remain unclear, particularly in resource-poor communities. We use a dynamic compartment model to assess GN-ARB transmission quantitatively, including the susceptible, colonised, infected, and removed populations at the community-hospital interface. We used two side streams to distinguish between individuals at high- and low-risk exposure to community ARB reservoirs. The model was calibrated using data from a cross-sectional cohort study (N = 357) in Chile and supplemented by existing literature. Most individuals acquired ARB from the community reservoirs (98%) rather than the hospital. High exposure to GN-ARB reservoirs was associated with 17% and 16% greater prevalence for GN-ARB carriage in the hospital and community settings, respectively. The higher exposure has led to 16% more infections and attributed mortality. Our results highlight the need for early-stage identification and testing capability of bloodstream infections caused by GN-ARB through a faster response at the community level, where most GN-ARB are likely to be acquired. Increasing treatment rates for individuals colonised or infected by GN-ARB and controlling the exposure to antibiotic consumption and GN-ARB reservoirs, is crucial to curve GN-ABR transmission.

## Introduction

The emergence and spread of antimicrobial-resistant microorganisms, particularly gram-negative (GN) antibiotic-resistant bacteria (ARB), affect population health globally^1–3^. Infections due to ARB are associated with significant disease burden, including higher mortality, longer hospitalisations, and increased health cost^4,5^. The worldwide emergence of resistance has occurred primarily due to antibiotic misuse and overconsumption^6^. The situation is even more critical in low-and middle-income countries (LMICs), where often no prescription is needed, and lack of access to novel compounds is common^7–9^. Moreover, the increase in demand for livestock products in LMICs has resulted in intensive use of antimicrobials in animal production, leading to an increase in ARB^10,11^. According to the Organization of Economic Cooperation and Development (OECD), the higher burden of GN-ARB faced by LMICs is associated with various factors including weaker regulations on the food industry and antibiotic use in humans or animals, inadequate water, sanitation, and hygiene infrastructure, and high contamination and concentration of environmental pollutants^12–14^.

GN bacteria impose a higher risk to public health than gram-positive pathogens, as they develop ARB faster, and there are fewer therapeutic alternatives available to manage these infections^15–17^. GN bacteria harbour a myriad of mechanisms to avoid antibiotics' action on their cell structure, such as the presence of degradational enzymes, efflux pumps, and membrane permeability^18^. The danger is imminent when facing infections caused by these pathogens, and particularly when dealing with bloodstream infections (BSI). Estimates suggest that BSI from ARB causes 33,000 annual deaths worldwide, the majority of which are caused by GN bacteria^19^. Most BSIs occur in the healthcare settings, particularly in intensive care units (ICUs)^2,20^. However, ARB infections, including community-onset BSIs^21^, are becoming increasingly relevant at the community level due to various factors, including inadequate antibiotic use, clonal dissemination, and community transmission networks and reservoirs, such as crowded households and workplaces, and educational facilities^22^.

Using a One Health approach, researchers in the past decade have drawn attention to the elevated exposure to environmental sources of transmission as one of the most relevant factors for the emergence and spread of GN-ARB in the community^11,23–34^. There is an ever-more evident connection between ARB in humans and environmental risk factors such as the interaction with animals (pets and livestock), food production and pesticides, poor waste management, contaminated water, and living conditions including crowding, pollution, and inadequate water, hygiene, and sanitation infrastructure^13,35^. All of these pose a high risk of transmission and disease burden, especially amongst the most disadvantaged populations where daily life might be easily jeopardised^36,37^. Furthermore, the existing disparate within-country health and sociodemographic inequalities in LMICs aggravate the situation. Indeed, there is a sizeable variation in ARB levels due to the context-specific high (hotspots) or moderate/low transmission risks within rural and urban settlements^38^.

The transmission of ARB and its human population dynamics have been modelled at the community level via SEIHS-adjusted (Susceptible, Exposed or colonised, Infected, and Hospitalised populations) compartmental models looking at in-human colonisation and infection^24,25,27,39–45^. Most existing literature considers antibiotic consumption as the primary driver of ARB^39–41^. However, recent population-based studies have examined interactions between healthcare settings and communities, suggesting that ARB are primarily acquired in the community when there is a significant presence of ARB reservoirs and, therefore, high transmission risks^25,27^. To the best of our knowledge, no modelling framework has considered disadvantaged populations, which account for about 80% of the global population, and this study aims to do that.

**Figure 1:**
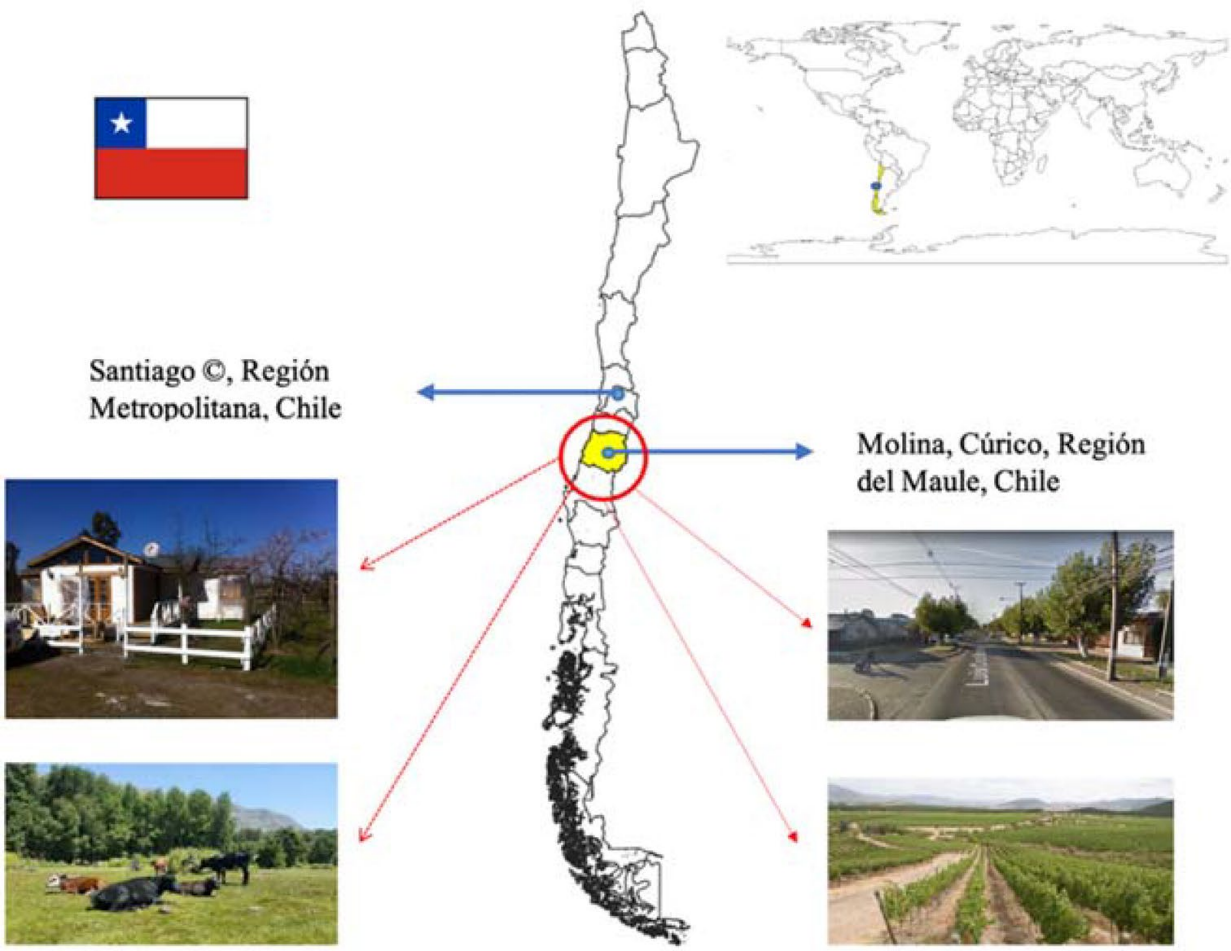
Study location. Notes: © stands for the capital city. Maule region is located in Chile's central-southern zone, where the rural-agricultural town of Molina is situated.

Specifically, we used a compartmental-based dynamic mathematical model to quantitatively characterise the dynamics of the transmission of GN-ARB in a rural community by looking at high and low risks of exposure to ARB reservoirs. We focused on Molina, a resource-poor peri-urban agricultural community in the south of Chile (Fig. 1). Previous research has shown a high prevalence of GN-ARB, specifically 40%, 29%, and 21% for quinolones-, third-generation cephalosporins-resistant, and carbapenem-resistant GN bacteria, respectively^46^. These estimated prevalence are substantially above OECD’s estimates for Chile (32%, 28%, and 17%, respectively)^14^. Consequently, we examined low and high GN-ARB transmission risk scenarios to help inform interventions to reduce the emergence of GN-ARB in a resource-limited community setting.

## Results

### Human population analysis

**Figure 2:**
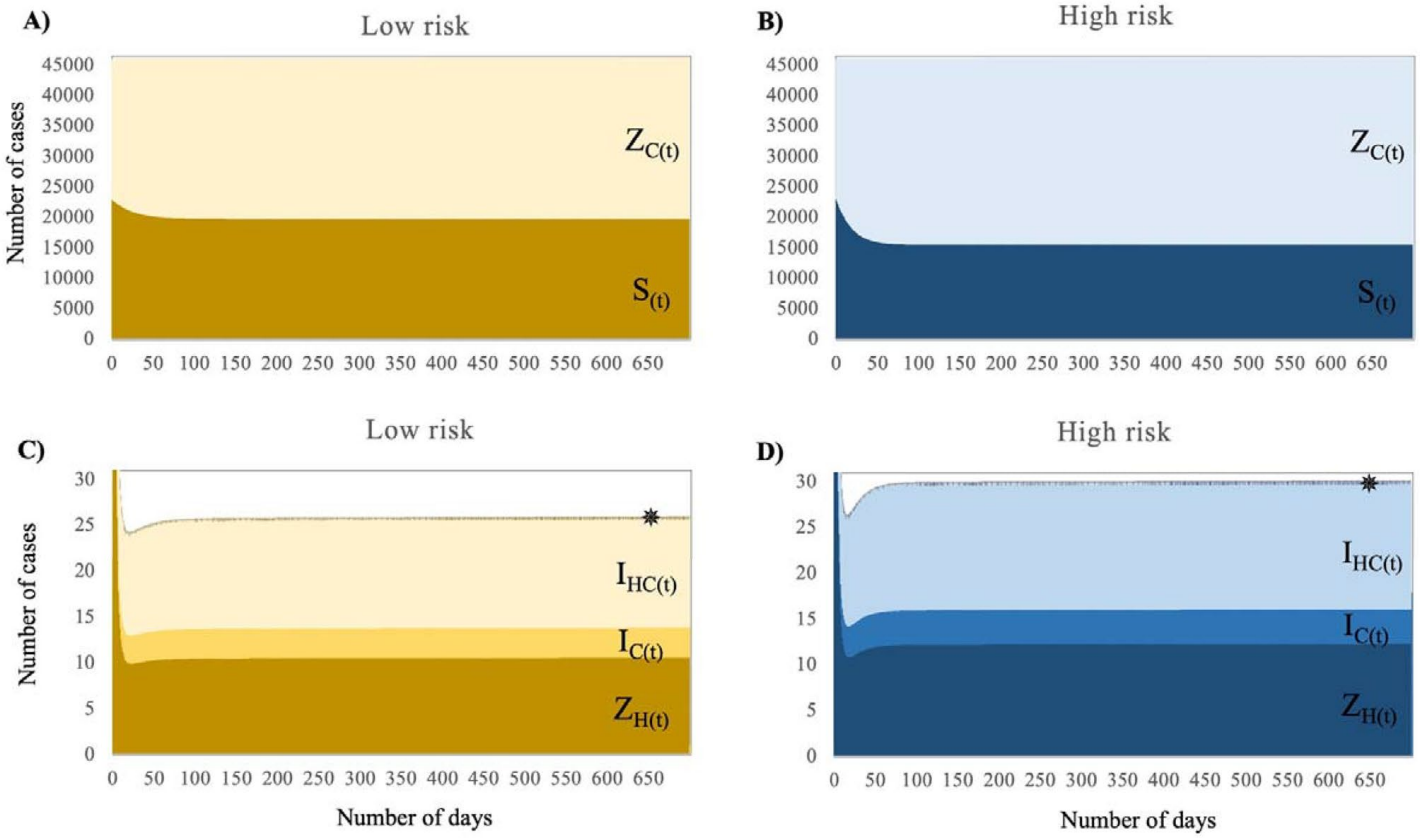
Population dynamics for individuals colonised or infected in the community or the hospital, by risk scenario. Notes: Panels (**A**) and (**B**) show the susceptible and colonised individuals in the community in the low-risk and high-risk scenarios, respectively. Panels (**C**) and (**D**) show individuals colonised at the hospital, or infected in the community or the hospital in the low risk and high-risk scenarios, respectively. ✵ stands for I_HH(t)._ Complete results of the population dynamics by compartment with their respective 95% CIs are presented in the supplementary material, Figs. D2-5.

Figure 2 shows the population dynamics for susceptible and colonised individuals in the community in the low-risk (panel A) and the high-risk (panel B) scenarios, and individuals colonised at the hospital, or infected in the community or the hospital in the low risk (Panel C) and high-risk (Panel D) scenarios. Our results suggest that ARB was primarily acquired in the community with a deficient proportion of inpatients with hospital-acquired GN-ARB colonisation and infection (Fig. 2, and Figs. D1–D5, supplementary material). However, the numbers increase under high exposure to ARB reservoirs within the community and hospitalised individuals. Even though transmission rates within the community are substantially lower than in the hospital, the entry rate of individuals into the hospital is meaner. Therefore, the model suggests that the dissemination of pathogens occurs vastly in the community posing a greater number of colonised individuals and disease burden due to the higher population size and direct/indirect person-to-ARB reservoirs contact. Our model suggests that 98% of the total GN-ARB acquisition (colonisation) occurs in the community, compared to 1% in the hospital, for the high- and low-risk scenarios. Even though transmission constitutes greater absolute numbers in the community, the percentage of people having hospital-acquired BSIs (H_HH_) from those colonised by GN-ARB within the hospital (Z_H_) was 3.36%, compared to 0.06% for the same definitions within the community (i.e., [I_C_ + I_HC_]/Z_C_). Finally, community-acquired infections (I_C_ and I_HC_) represented 97.6% of the total population of individuals having BSIs; however, the community mortality burden was low (7.8%), compared to the hospital (92.2%) where most individuals are treated for BSIs.

### Population dynamics by risk-scenario

**Figure 3:**
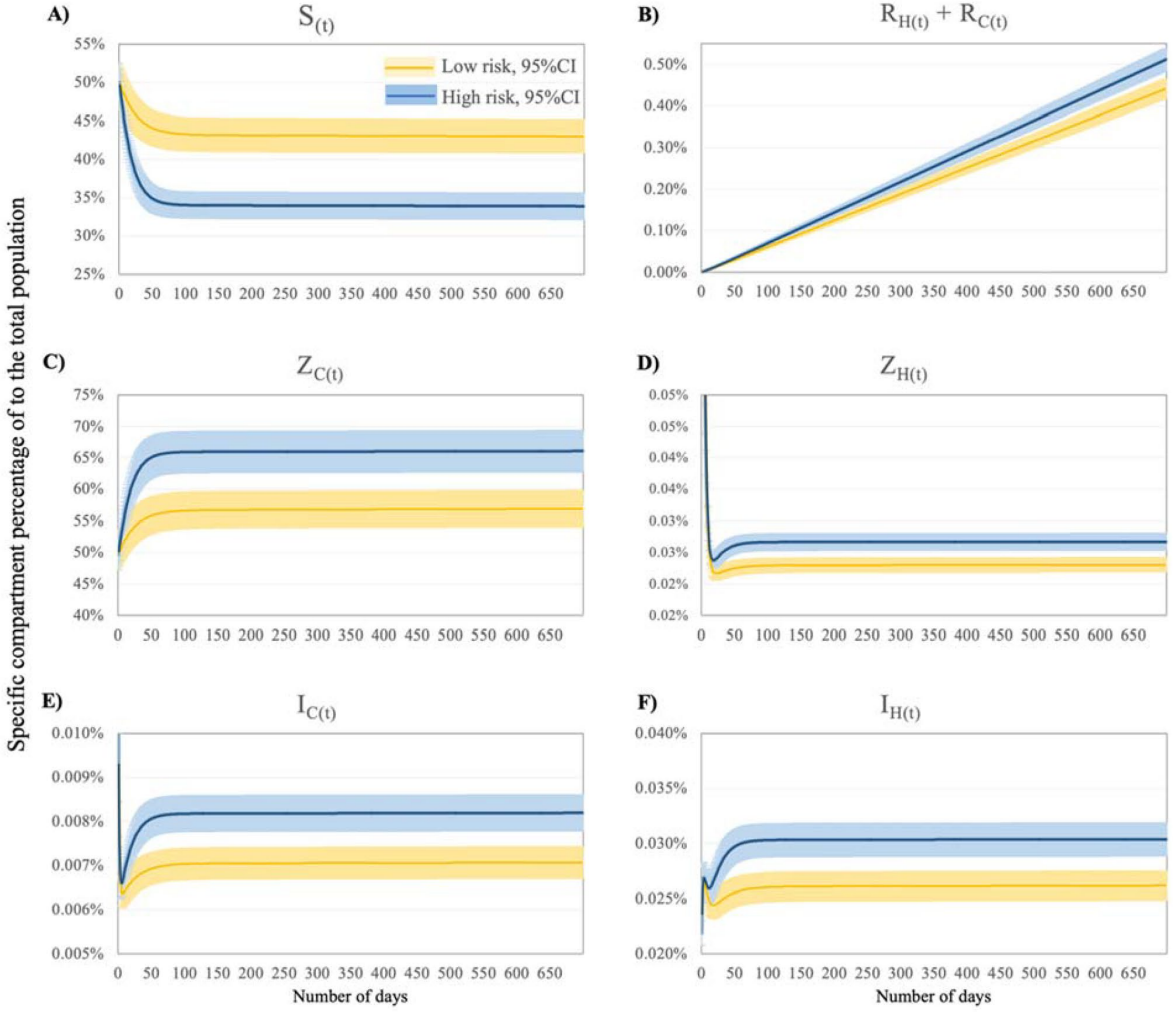
Proportion of the population (N) per compartment and overtime, by risk group. Notes: All groups coloured in light blue (or dark) sum up 100% of the population (N) including those removed. The proportions were calculated from the main model over the end of the study period (also see Fig. D5, supplementary material). S: Susceptible population, Z_C_: Colonised individuals by a GN-ARB in the community; R_C_: Individuals removed (dead due to GN-ARB BSI in the community); R_H_: Individuals removed (dead due to GN-ARB BSI in the hospital). I_H_: Individuals with a GN-ARB BSI in the hospital, that is comprised of I_HH_: Individuals with a hospital-acquired GN-ARB BSI; I_HC_: Individuals with a community-acquired GN-ARB BSI; I_H_: Individuals with a GN-ARB BSI in the community; Z_H_: Colonised individuals by a GN-ARB in the hospital. Figure D5.2 (supplementary material) shows the proportion of the total population by compartment and risk scenario at the end period. Y-axes stand for each specific compartment proportion to the total population (e.g., the y-axis in panel A stands for the proportion of the total population consisting of S(t)).

Figure 3 depicts the proportion of the total population (N) over time, by compartment and risk scenario (see Fig. D5, supplementary material for model specific results at the end of the study period). Compared to the low-risk scenario, the proportion of susceptible population rapidly decreased under the high-risk scenario posing a higher disease burden (Fig. 3, panel A). Higher exposure to ARB reservoirs (27% greater derived from the $\rho$ coefficient) translates into an increased proportion of Z_C_ under the high-risk scenario, which exceeded the low-risk comparison group by 10 raw percentage points (16% higher population size; see Fig. 3 panel C). Consequently, the susceptible population was reduced by 21% after contrasting both groups (e.g., {Z_C High-risk_ − Z_C Low-risk_}/Z_C Low-risk_). The rest of the compartments presented less than 2% of the total population in the system modelled (Fig. 3, panel B, D, E and F). Compared to the low-risk scenario, the results show a significant variation over the number of hospitalised individuals colonised by GN-ARB (Z_H_ was 17.4% higher, Fig. 3 panel D), hospital- and community-acquired infections (I_HC_ and I_HH_ were 16.1% and 16.0% higher, respectively), and deaths (removed) (15.9% greater) in the high-risk scenario (Fig. 3, panel B).

### Sensitivity analyses

Our sensitivity analyses showed that the most influential parameters increasing GN-ARB transmission were hospitalisation rate (${\delta }_{I}$), treatment rate for hospitalised individuals having BSIs (${\omega }_{H}$), and the probability of a having a GN-ARB BSI in the community (${\xi }_{C}$). Diversely, the probability of having a GN-ARB BSI in the hospital (${\xi }_{H}$) was one of the less influential parameters determining GN-ARB transmission.

In univariate analyses, we observed that a 0.1 increase over the hospitalisation rate (${\delta }_{I}$=0.9, compared to baseline ${\delta }_{I}$=0.8) produced 9% and 10% fewer GN-ARB BSIs within the community under high and low-risk scenarios, and 1.6- and 0.6-times fewer deaths attributed to GN-ARB BSIs, respectively (Fig. D6.1–2, supplementary material). Moreover, varying the probability of having a BSI in the community (${\xi }_{C}$) had a directly proportional effect (linear) on the number of BSIs in the community and hospital settings (i.e., two times higher ${\xi }_{C}$ is translated into two times greater number of individuals having BSIs) and the attributable number of deaths, regardless of the risk scenario (Fig. D7.1–2, supplementary material).

Conversely, increasing (${\xi }_{H}$) had a negligible effect on the number of hospital-acquired infections and no effect on the total number of infections within the hospital (including community- and hospital-acquired infections; Fig. D8, supplementary material). Similarly, improving treatment rates by 25% (${\omega }_{H}$) within the hospital setting reduced the prevalence of hospital-acquired infections by 19% under both risk scenarios (Fig. D9, supplementary material). Our results also showed that larger clearance rates ($\gamma$) support bacterial decolonisation, highly favouring the low-risk scenario (e.g., a 25% improvement over $\gamma$ produced a 15.5% decrease in BSIs in the community, compared to 13.5% under the high-risk scenario: Fig. D10, supplementary material).

Finally, we analysed different hypothetical cases for the transmission parameter ($\beta$_C_) which highly impacts the susceptible and colonised populations (Fig. D11, supplementary material). Greater transmission rates had a non-substantial effect on the crude numbers of individuals having BSIs (six to eight more individuals with BSIs observed if $\beta$_C_ is ten times greater). Even though these crude numbers are not significant in magnitude, we noted that higher $\beta$_C_ is associated with a higher marginal increase of the number of BSIs under the low risk than the high-risk scenario. For example, a 25% higher $\beta$_C_ is translated into a 16.6% and 10% greater number of individuals with BSIs in the hospital, respectively.

**Figure 4:**
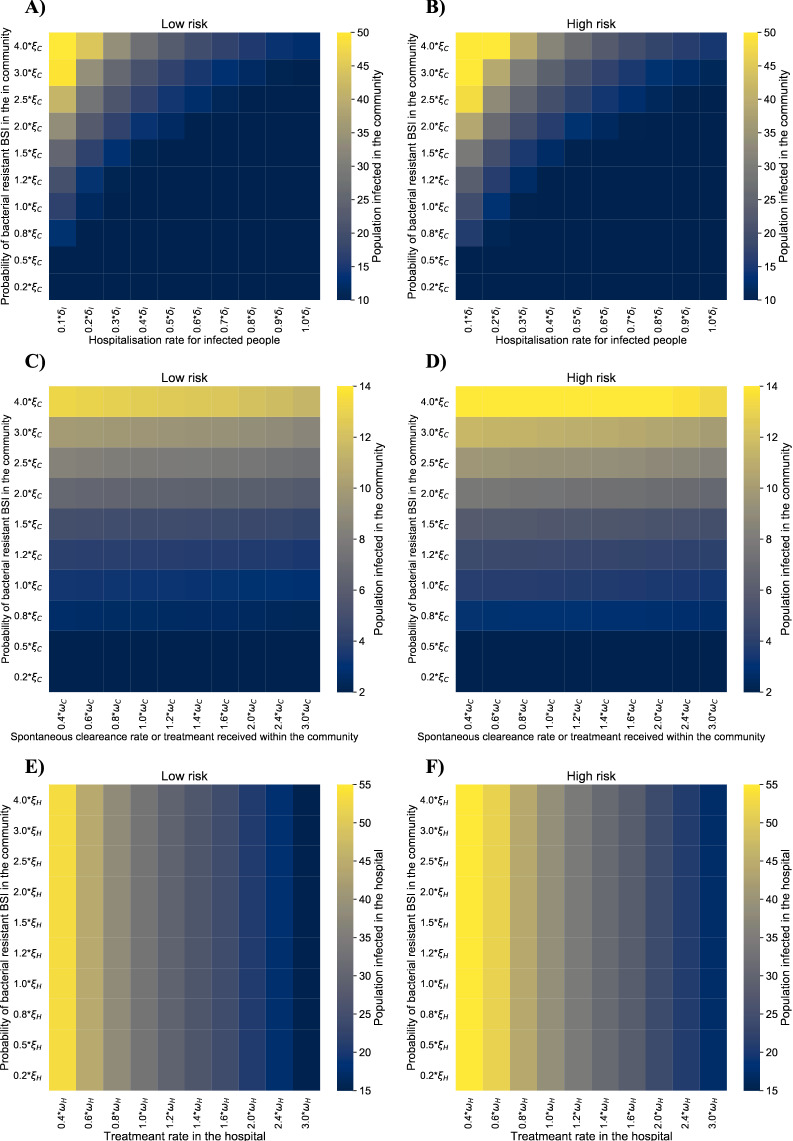
Bivariate analyses result of the main parameters on the number of infections in the community (panel **A**–**D**) and hospital (panel **E**–**F**), by risk group. Notes: A and B show a bivariate analysis of the hospitalisation rate for individuals with a GN-ARB BSI and the probability of developing GN-ARB BSI, both in the community, on the number of individuals having GN-ARB BSI in the community. Figures (**C**) and (**D**) depict the variation of the probability of GN-ARB BSI and the spontaneous clearance (or treatment rate), both in the community. Figures (**E**) and (**F**) display the bivariate relationship between the probability of GN-ARB and treatment rate, both in the hospitals, on the number of people having GN-ARB in the same setting. Lighter (yellow) colours mean a higher number of people infected by GN-ARB, whereas darker (blue) colours mean lower.

In the bivariate analyses, we found that individuals having community-acquired BSIs would be reduced to the minimum (15 cases on average) if hospitalisation rates for people with BSIs are improved or in the range of 60% < ${\delta }_{I}$<100% and ${\xi }_{C}$ is diminished or between 0.2*${\xi }_{C}$ and 1.5*${\xi }_{C}$ (Fig. 4, panels A and B). ${\xi }_{C}$ is directly related to the greater number of community-acquired even if profound changes are seen over the spontaneous clearance or treatment rate received for BSIs within the community (${\omega }_{C}$) (Fig. 4, panels C and D). However, when it comes to hospitalised patients, the treatment rate for BSIs (${\omega }_{H}$) directly affect the number of individuals with BSIs (greater impact over community-acquired), despite of ${\xi }_{H}$, and displaying the highest burden for those individuals at high-risk (Fig. 4, panels E and F).

In antibiotic-specific models, we found a 9.9% higher percentage of people colonised and infected by GN quinolone-resistant bacteria in the community over time, compared to our base GN-ARB model for the high-risk group (Fig. E5, bottom panel). Whereas it was 1.6% greater for cephalosporins-resistant GN bacteria, and 13.4% lower for carbapenem-resistant GN bacteria. Among the different bacterium transmission parameters surveyed from the literature, we identified minor variations after accounting for the Enterobacterales family and other than Enterobacterales, including *Acinetobacter baumanii*, specific transmission dynamics (observed variation < 0.0017% compared to our base GN-ARB model; supplementary material, Fig. E6).

**Figure 5:**
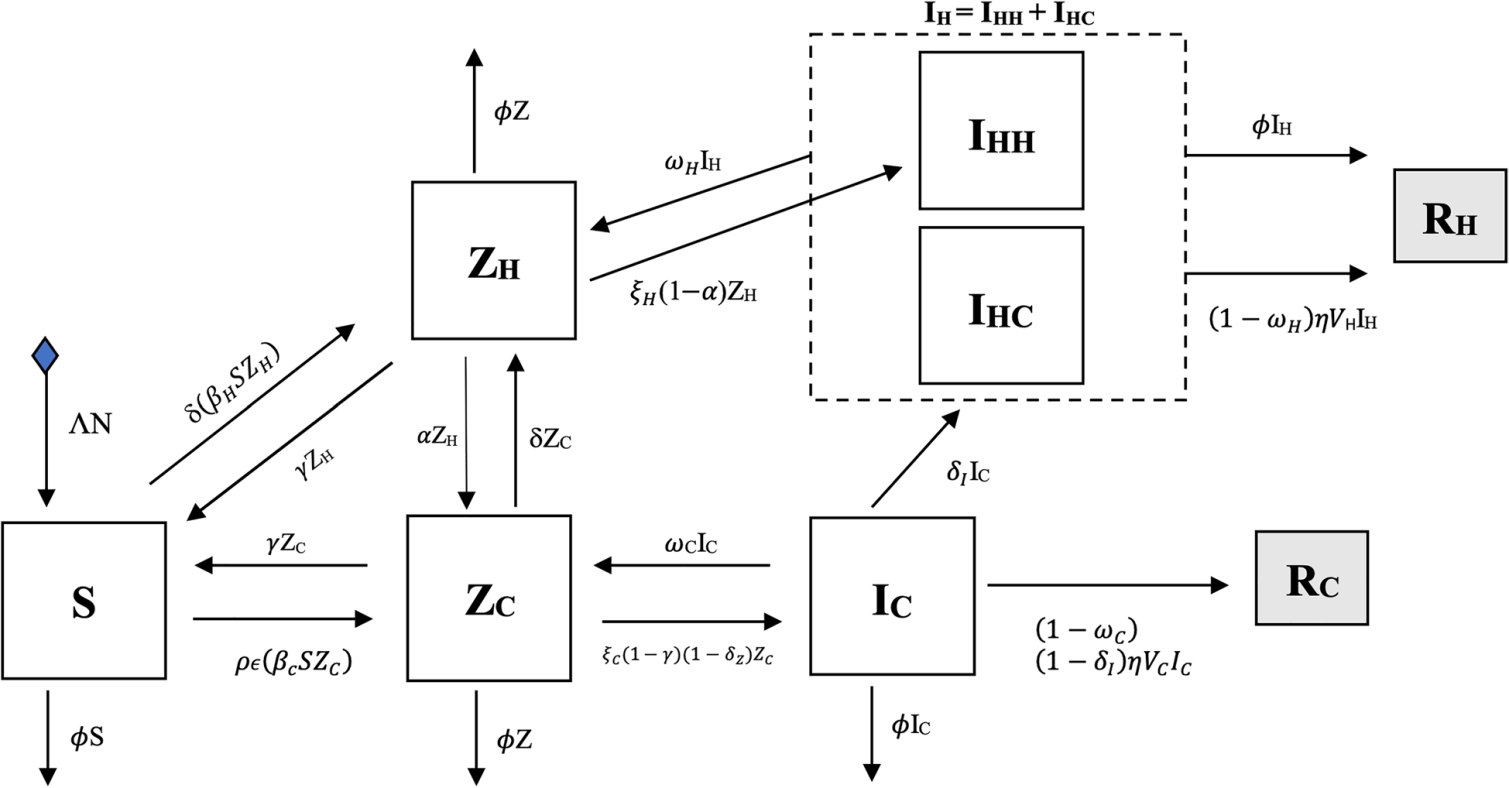
Compartment model for GN-ARB community transmission. Notes: Tables 1 and 2 show baseline conditions and parameter specifications of the compartment model. Subscript “H” stands for hospital population whereas “C” for community. I_H_ is divided into I_HH_ and I_HC_ for hospital- and community-acquired BSIs. The compartment S indicates that the whole cohort is susceptible to antibiotics because gram-negative bacteria are an essential part of human gut microbiota and other mucosal surfaces. Infectiousness (I) occurs after being colonised (Z) by resistant bacteria. Individuals are immediately transferred to the susceptible S(t) disease-free population compartment after full clearance of infection. The model assumes that people can be infected only by one type of resistant bacterium simultaneously, which cannot evolve. (R) compartments are for removed individuals (death).

## Discussion

Our results suggest that most GN-ARB acquisition (colonisation) is observed in the community, causing a substantial impact on the number of infections and mortality, particularly among those individuals at high-risk exposure to ARB reservoirs. This result is consistent with the literature on transmission dynamics in disadvantaged and rural communities, where inadequate access to clean water, sanitation, and hygiene infrastructure and higher exposure to pollution and GN-ARB reservoirs impose a considerable risk to the health system and community health^47–50^.

Similar to a recent study^25^, most of the human acquisition of ARB is related to the large number of people agglomerated in community settings (~ 98% of the total population), where close contact is common, including crowded households, educational facilities, and agricultural workplaces. For instance, the turnover of the hospital population in Molina was about 0.000054 per day, according to the Chilean Ministry of Health^51^, which evidences the reduced patients’ inflow. Our study expands from previous modelling studies^24,27,45,52,53^ in two ways. Firstly, it includes the acquisition of ARB using a broader population perspective while incorporating the hospital and community populations from a recently studied rural middle-income community. This is in contrast to most of the existing mathematical models, which are largely focused on high-income urban areas. Secondly, we generate two risk scenarios for community reservoirs and analyse the contribution of ARB carriage and burden associated, which differs from previous efforts that mainly analyse hospital population dynamics and transmission risks in that particular setting^26,27,42–44^.

### Why does the high-risk scenario pose a greater exposure to ARB reservoirs?

Compared to the low-risk scenario, the model results showed that the high-risk scenario resulted in 1.27 times higher ARB rate due to higher exposure to GN-ARB reservoirs or risk factors in the community, consistent with the literature^30,54–61^. We found that high-risk exposure exacerbated the disease burden produced by GN-ARB in the community and hospital. The main risk factors in the community were previous antibiotic consumption and overcrowded spaces. Increased community-level antibiotic consumption has been associated with inappropriate antibiotic dispensing and misuse^54^, specifically in rural and most deprived areas where poor health determinants exacerbate the development and acquisition of resistant strains^55^. Crowded households and higher animal contact increase the exposure of susceptible populations to colonisation with ARB^56^. However, other local factors previously documented in Molina might influence these figures secondarily^60^. For instance, antibiotic consumption in food-producing animals in Chile has been estimated at 77 mg per animal population unit (cattle, pigs, or chicken)^11^ with 24.8% ARB prevalence among them^58^, posing greater risks at the human-animal interface and specifically to rural agricultural areas such as Molina^57^. Also, the high mass of agriculture-related workers who are frequently exposed to animal contact and chemical hazards (e.g., toxic substances, fertilisers, and pesticides) may result in selective pressure that promotes the development of antimicrobial resistance among specific bacterial populations^59^.

### Sensitivity analyses

Our model's key parameters that modify ARB acquisition and infections are the transmission parameter, hospitalisation rates, exposure to ARB reservoirs, and bacterial spontaneous clearance rates. Whereas higher treatment rates within hospitalised individuals and probability of developing community-acquired BSIs increased considerably the number of infections and mortality burden in the hospital. ARB carriage could be mitigated controlling transmission rates (e.g., 25% increase in β indicates 15.5% higher number of individuals colonised by GN-ARB), which affect the number of infections in the community (12% increase) and mortality.

If appropriate healthcare for GN-ARB BSIs was improved at the hospital, the number of subsequent infections would be substantially lower regardless of the risk scenario. A two-fold improvement in targeting bacterial clearance and treatment in the community would reduce colonisation and infection rates in the community and hospital settings by decreasing the prevalence of BSIs by 50–75%, with a higher impact in the low-risk scenario (75% decrease).

Quinolone-resistant GN bacteria posed the greatest burden, according to our study results. A 9.9% higher prevalence of colonised and infected people was observed compared to our base GN-ARB model results. Quinolones were introduced in Chile between 1998 and 2015, primarily for veterinary usage, including aquaculture and agriculture^62^. Recent reports showed excessive use and resistance genes among residues in marine sediments, which has contributed to a larger number of human pathogens isolated in those areas^63^.

### Interventions and policy

Firstly, if reducing ARB colonisation is our goal, interventions should target exposure in the community. Previous studies have described interventions that may be useful locally to reduce transmission and carriage^64–66^. Through awareness campaigns by employing large-scale educational and stewardship programs, changes in human behaviour may decrease the spread of GN-ARB in the community^64^. In particular, the development and guidance of prescription standards to avoid unnecessary consumption (overconsumption and misuse) and over-the-counter sales of antibiotics is paramount^27^.

It is essential to prevent new cases through active community surveillance programs and mitigate the potential risks of environmental and hospital exposures, including antibiotic use in food-producing animals and contaminated food^61,65–67^. Applying a One Health approach may help control the transfer of ARB genes at the animal-human interface (direct or indirect contact with livestock or food chain), because clonal dissemination plays an important role in the spread of ARB pathogens. Research on the specific transmission mechanisms is urgently needed to design more effective interventions to reduce ARB carriage in humans^68^. Less acquisition of ARB in the community and hospital settings is also facilitated with higher rates of clearance, suggesting that community-based (or post-discharge) decolonisation schemes would bottom down ARB prevalence shifting the transmission dynamics over time. For instance, improving the adherence to hand hygiene has been cost-effective in reducing the acquisition of GN-ARB (carriage) by 10% in a 6-month period^69^. Additionally, universal screening for incoming patients has been cost saving (compared to doing nothing) to reduce ABR carriage in hospitals where ARB incidence levels are above 0.3%^70^, which represents the case of the community hospital examined in the present study.

Secondly, if reducing mortality attributed to GN-ARB BSI is the focus, increasing healthcare access and quality (enhanced accessibility and active surveillance) and improving treatment success rates for BSIs within the hospital are critical. In particular, early-stage identification (and testing capability) through a rapid response from healthcare facilities might favour the early prediction of the clinical progression of BSIs in communities at high risk right before the disease burden is aggravated^71,72^. The risk of BSIs is notably greater in low-and-middle-income communities due to under-resourced infrastructure to diagnose and quantify these complications. Current standards to detect GN-ARB BSIs in hospitals are limited to automated blood culture systems within laboratories having reduced capacity, which may take up to 2–4 days^73^. New rapid diagnostic assays are available for identifying and detecting the causative organism and its susceptibility, producing earlier outcomes than conventional phenotypic susceptibility and subculture testing^74^. However, their high cost is frequently a major hurdle to their implementation in low-resource settings such as Molina.

### Limitations, assumptions, and strengths of the study

The article has some limitations. First, there is a coexistence of drug-susceptible and drug-resistant GN bacteria calibrated to existing data and literature parameters that are pathogen-specific, which might differ from less transmissible pathogens and organisms’ aetiology. However, no other community-based study provides information on ARB for other pathogens-ATB combination pairs in Chile. Second, our model was calibrated based on a small sample of study participants. This may affect the model results because of the high variability in the computation of some of the parameters, potentially introducing biases. Also, age-specific or sociodemographic stratification was not included due to sample constraints. Nevertheless, this is the first study in a middle-income community where ARB was tested by collecting and analysing faecal samples for GN-ARB colonisation in humans. Third, literature is absent on GN-ARB in most disadvantaged communities, so the main parameters were primarily obtained from studies focusing on middle and high-income countries. This may have underestimated the effects over population dynamics, but no further evidence is available, and data were provided from high-quality and reliable sources.

We assumed spontaneous clearance of ABR was equivalent among individuals in the community and the hospital. However, clearance rates might be higher in the community because hospitalized patients are ill or may have weaker immune systems.

One critical parameter is the hospitalisation rate for people with GN-ARB BSI in a rural community because it is highly sensitive to the prevalence of untreated BSI. We assumed this to be 80% considering that BSI required immediate attention^75^. This parameter could be lower in low-income settings with limited access to healthcare, but we did not have data to test this assumption. Our estimates should be considered conservative; if hospitalization rates were lower, the disease burden would be even higher than estimated.

We assumed that transmission rates in the community are lower than in the hospital, but that ARB reservoirs exposure is higher. For instance, our study suggests that greater consumption of β-lactam antibiotics, which have been incrementally introduced in the community^46^, might be associated with a higher prevalence of community-acquired ARB acquisition. This may change if other pathogens are introduced into the community. For example, co-colonization with clinically relevant ARB-pathogens might occur, favouring horizontal gene transfer and further dissemination of ARB. However, we focused on the most likely scenarios to occur. Also, we could not perform pathogen-specific models because gram staining was primarily used in the parallel study. Nevertheless, we sourced transmission parameters for different GN-ARB types and did not observe meaningful variations over time.

Our study's main strengths include using a novel approach to account for the high risk of exposure to GN-ARB reservoirs in the community by considering their indirect interaction with the environment, hospitals, and animals as risk variables. We used an existing and transparent quantitative framework, including extensive sensitivity analyses, to cover a broader range of scenarios where people may acquire GN-ARB. Interventions are also suggested depending on the problem to be targeted. Specifically, antibiotic decolonisation in extensive therapies might be highly cost-effective in high-risk and limited-resourced scenarios to account for a reduced transmission and burden of disease in the future^76^. This study forms part of the evidence base required to prioritise new strategies to ease GN-ARB transmission and its associated burden, including using vaccines and diagnostics rollout as cost-effectiveness analyses to combat the short and long term spread^27,77^. Likewise, modelling approaches should consider stochastic effects in the modelling structure as most disadvantaged communities face constant economic and social fluctuations or a higher exposure to natural disasters (e.g., earthquakes, floods).

### Rethinking the surveillance system

Our study model incentives an integrated One-Health approach within the local surveillance program to tackle antimicrobial use and resistance in humans and animals, particularly in communities at higher risks of antibiotic exposure^68^. Multisectoral synergetic efforts between the plant and food safety, environmental sources and wildlife, and human and animal health departments could be promoted by the authorities and national ARB action plan to control the presence of ARB with active surveillance. Identifying and testing individuals in communities of highly endemic risk exposure should be prioritised by developing locality-specific evaluation protocols to target ARB dissemination and associated disease burden rapidly and effectively.

Our study lays out a different structure for ARB quantification and burden attributed by considering the impact of the community exposure to ARB reservoirs. We do not need to emphasize whether ARB is detected in the hospital setting, but rather target the community where ARB is acquired. Our results suggest that including GN-ARB control in the community will help stop further propagation, specifically for those individuals at high risk of ARB exposure scenarios. ARB in the communities is increasing; understanding and quantifying transmission is essential to curb this emerging public health problem.

## Methods

### Mathematical model and setting description

We developed a dynamic compartmental model for GN-ARB transmission in the rural town of Molina. Molina has 46,000 inhabitants^78^, a poverty rate of about 13.5%, and one of the highest age-standardized mortality rates for chronic diseases in Chile^79^. High income inequality, low economic growth, and a substantial proportion of the population with inadequate water, sanitation, and hygiene infrastructure, make Molina a heterogeneous and useful case study to understand GN-ARB transmission beyond western high-income countries (supplementary material, sections A and B).

Figure 5 shows the flow diagram of the compartmental model. We considered Molina’s population in time “*t*” (N(*t*)), and demography dynamics to be regulated by a constant birth rate (Λ) and a natural mortality rate given by ($\varnothing$). Henceforth, “bacteria” refers to GN bacteria. The model has eight compartments accounting for susceptible population S(t); population colonised by a GN-ARB either at the community Z_C_(t) or hospital Z_H_(t), which can be denoted as exposed population with no BSI; and population with bloodstream infections caused by resistant bacteria either at the community I_C_(t) or hospital setting I_H_(t). The latter is divided into those with hospital-acquired infections (I_HH_(t)) and community-acquired (I_HC_(t)). Finally, individuals might die due to GN-ARB BSIs in the hospital or community settings (R_H_ and R_C_, respectively).

We assumed that the population is constant N(t) = S(t) + Z_C_(t) + Z_H_(t) + I_C_(t) + I_HC_(t) + I_HH_(t) + R_C_(t) + R_H_(t) and the presence of GN-ARB determines colonisation. The disease is defined as BSI caused by GN-ARB. We employed the same model for high and low exposure to ARB reservoirs (risk groups/scenarios) in the community. Our model was initiated using baseline data from late 2018 and early 2019. Differential equations are shown in the supplementary material, section C.

### Data collection and measurement specifications

Baseline conditions within the compartments and the risk coefficient for ARB reservoirs exposure were extracted from a previous study^80^. The study provides the prevalence of GN-ARB and the exposure to ARB reservoirs in the Chilean community between 12/2018 until 5/2019. GN-ARB included any quinolone-resistant, extended-spectrum cephalosporin-resistant, or carbapenem-resistant GN bacteria. Gram staining was used to differentiate GN bacteria. The prevalence of GN-ARB in the hospital was also incorporated, based on the same antibiotic-bacterium pairs. Section A of the supplementary material contains further details on the study used to extract our data and parameters’ information.

### Model parametrisation

Tables 1 and 2 show the initial conditions and the description of the main variables in the model. We calibrated the transmission parameter ($\beta$) by matching the model projected incidence to the overall incidence of GN-ARB colonisation over time provided from a longitudinal study of bacterial susceptibility levels (incidence) to ATB within Chilean hospitals^81^ using an Approximate Bayesian Computation Markov chain Monte Carlo simulation^82^. To compute $\beta$, we tested different regression specifications (e.g., linear, Gaussian, and polynomial) to get the best goodness-of-fit based on R^2^. Consequently, as $\beta$ was calibrated to hospital data, we used a population-based ratio obtained from the literature^25^ to adjust the parameter to the community. Our risk coefficient ($\rho$) indicates a high-risk scenario of exposure to ARB reservoirs, compared to a low-risk scenario. It was computed following a two-stage protocol. Firstly, we employed a logistic regression to capture the adjusted ARB rates, obtained from the parallel study on colonisation of GN-ARB in the community. Then, we divided the predicted ARB rates from the previous step into two groups: high and low ARB (calculated as predicted adjusted rates above or below the median values, respectively). Secondly, we calculated the risk ratios using a 2 × 2 matrix between high/low ARB groups and the variable of high/low exposure to ARB reservoirs in the community (ϱ). Community ARB reservoirs considered control variables for the computation of ρ were antibiotic consumption, animal proximity, and contact, household overcrowding, animal products consumption, agricultural occupation for exposure to pesticides, previous hospitalisation, and other sociodemographic variables. All details on the computation of these parameters are found in supplementary material, section B.

**Table 1:** Baseline conditions within the compartments.

Symbol	Description [Units]	Baseline value	Source
N(t)	Population size [nº individuals]	N_(t=0)_ = 46,000	DEIS, Chilean Ministry of Health ^51^
S(t)	Susceptible individuals to GN-ARB [nº individuals]	S_(t=0)_ = 22,936	MAUCO and MDR-GN studies ^46,79^
Z_C_(t)	People in the community colonised by a GN-ARB [nº individuals]	Z_C(t=0)_ = 23,064 − Z_H(t=0)_ − I_C(t=0)_ − I_HC(t=0)_ − I_HH(t=0)_	MAUCO and MDR-GN studies ^46,79^
Z_H_(t)	People in the hospital colonised by a GN-ARB [nº individuals]	Z_H(t=0)_ = H*(1–0.0842)	(1–8.42%) individuals are colonised in the hospital and not yet infected. H was calculated in the supplementary material
I_C_(t)	Individuals in the community infected by a GN-ARB [nº individuals] ^c^	I_C(t=0)_ = I_H(t=0)_*0.5	Assumed at baseline
I_HC_(t)	Individuals in the hospital having a bloodstream infection (community-acquired) caused by a GN- who attended the local hospital [nº individuals] ^c^	I_HC(t=0)_ = H*0.0842*0.83	83% of individual in the hospital having a bloodstream infection ^83^
I_HH_(t)	Individuals in the hospital having a bloodstream infection (hospital-acquired) caused by a GN- who attended the local hospital [nº individuals] ^c^	I_HC(t=0)_ = H*0.0842*0.17	17% of individual in the hospital having a bloodstream infection ^83^

DEIS: Dirección de Estadísticas en Información en Salud; GN-ARB: gram-negative antibiotic resistance bacteria; MDR-GN: Multidrug resistant gram-negative, OECD: Organisation for Economic Co-operation and Development. Parameters were obtained from the literature and available resources from the Chilean government. Parameters follow the structure of Fig. 5 and Equations from the supplementary material. ^c^ We used the corresponding proportions for community and hospital-acquired infections from a study in the USA ^83^. Chile and the USA have similar ARB rates according to OECD estimates ^14^.

**Table 2:** Parameters of the compartment model for GN- transmission.

Parameters	Description [Units]	Baseline value	Source
Λ	Birth rate [annual number of new-borns/population size/365 days]	0.000032^a^	DEIS, Chilean Ministry of Health ^51^
$\varnothing$	Death rate [annual number of all deaths/population size/365 days]	0.000019^b^	DEIS, Chilean Ministry of Health ^51^
${\xi }_{C}$	Probability of a bacterial resistant bloodstream infection to occur in the community [%]	${\xi }_{H}$/100	We used a comparison ratio described elsewhere^25^
${\xi }_{H}$	Probability of a bacterial resistant bloodstream infection to occur in the hospital [%]	0.011	Incidence of BSI^84^ for GN- in the community, based on OECD standards for *E. coli, P. aeruginosa* and *K. pneumoniae*^14^
${V}_{C}$	Disease-induced drug-resistant mortality rate for people with BSIs in the community [1/unit time] [%]	0.368	^84^
${V}_{H}$	Disease-induced drug-resistant mortality rate for patients with BSIs in the hospital [1/unit time] [%]	${V}_{C}$/1.31	^25,85^
$\alpha$	Rate at which those in the hospital return to the community	0.32	^25^
$\gamma$	Spontaneous clearance of colonisation [%]	1/42 per day	^86^
$\beta$_H_	Overall transmission rate for GN-ARB in the community [coefficient]	0.0005308	Estimated using hospital data ^81,87^ See supplementary material, section B
$\beta$_C_	Overall transmission rate for GN-ARB in the hospital [coefficient]	$\beta$_H_*0.25 ^c^	Using $\beta$_H_ and comparison ratio from the literature for the relationship between hospital and community transmission ^25^
${\omega }_{H}$	Spontaneous clearance of a bloodstream infection caused by a GN-ARB, and or treatment received to eliminate it in the hospital [%]	0.2 per day	^44^
${\omega }_{C}$	Spontaneous clearance of a bloodstream infection caused by a GN-ARB, and or treatment received to eliminate it in the community [%]	0.25*$\omega$_H_	We assumed spontaneous clearance or treatment received is less likely to occur in the community compared to the hospital
$\eta$	Average duration of the bacteremia in days until people either are removed or recovered	0.10	^88,89^
$\rho$	Low/High-risk coefficient for transmission in people facing a low/high exposure to ARB reservoirs [coefficient]	1 and 1.27	Estimated for low and high-risk scenarios See supplementary material, section B
$\delta$	Hospitalization rate for people in the community [%]	5.4 × 10^–5^	Estimated See supplementary material, section B
${\delta }_{I}$	Hospitalization rate for people with community-acquired BSI caused by a GN-ARB [%]	0.8	Assumed
$\epsilon$	Antibiotic exposure in the community [coefficient]	0.01253	DDD per 1000 inhabitants (12.53) extracted from ^90^

DDD stands for defined daily dose; DEIS: Dirección de Estadísticas en Información en Salud; GN-ARB: gram-negative antibiotic resistance; OECD: Organization for Economic Co-operation and Development. ^a^[542/45,976]/365. ^b^[314/45,976]/365. ^c^0.0005308*0.25.

We utilised Monte Carlo simulations to estimate 95% confidence intervals (CI) for each compartment to account for uncertainty in decision-making and quantitative risks. We estimated the 95% CI by recalculating $\beta$ based on a random normal distribution and using 1000 replications.

Additionally, we carried out sensitivity analyses over the main parameters to ensure the results did not hinge on parameter assumptions. GN-ARB BSI probability and hospitalisation rate in the community are the primary sources of uncertainty over the disease dynamics of GN-ARB. We employed univariate and bivariate sensitivity analyses over the main parameters associated with higher GN-ARB burden to account for this variability. One way sensitivity analyses included the variation over the probability of BSI in the community ${(\xi }_{C})$ and the hospital (${\xi }_{H})$, and the rate of hospitalization for GN-ARB infections ${(\delta }_{I})$ as it is unclear how many people get hospitalised for BSI in a rural community. We used a two-way sensitivity analysis for the GN-ARB BSI probability and hospitalisation rate in the community and GN-ARB BSI probability and treatment rate in the hospital setting due to the higher risk of developing hospital-acquired BSIs (e.g., from the use of intravenous devices, therapeutic interventions). Furthermore, we divided our model into antibiotic-specific resistance types, including GN resistant to carbapenems, cephalosporins, and quinolones. We adjusted our $\beta$ and ϱ parameters to the detailed antibiotics (supplementary material, section E). Also, we surveyed the literature to understand the magnitude of the transmission parameters following different GN bacteria type (supplementary material, Section E).

The complete analysis was computed on MATLAB ® version R2019b (MathWorks Inc., Natick, MA, USA www.mathworks.com) and Python programming language version 3.9 (Python Software Foundation, https://www.python.org/). The complete script is available on Python Jupyter notebook at https://bit.ly/2ZpucKh.

## Supplementary Information

Supplementary Information.

## Data Availability

Data is publicly available or within the manuscript. The complete script is available on Python Jupyter notebook at https://bit.ly/2ZpucKh. No ethics approval required.

## Abbreviations

ARB: Antibiotic-resistant bacteria
ATB: Antibiotic
BSI: Bloodstream Infections
GN: Gram-negative
HICs: High-income countries
ICU: Intensive care units
LMICs: Low- and middle-income countries
MDR: Multidrug resistance
OECD: Organisation of Economic Cooperation and Development
WHO: World Health Organisation
