# Supplementary material for: Transmission of gram-negative antibiotic-resistant bacteria following differing exposure to antibiotic-resistance reservoirs in a rural community: a modelling study for bloodstream infections
Source: Sci Rep. 2022 Aug 5;12:13488. doi: 10.1038/s41598-022-17598-x (PMC9356060; doi:10.1038/s41598-022-17598-x)
Supplement: Supplementary file 1 — Supplementary Information. [file 41598_2022_17598_MOESM1_ESM.docx]

**SUPPLEMENTARY MATERIAL**

Transmission of gram-negative antibiotic-resistant bacteria following differing exposure to antibiotic-resistance reservoirs in a rural community: a modelling study for bloodstream infections

Kasim Allel, Lara Goscé, Rafael Araos, Daniel Toro, Catterina Ferreccio, Jose M. Munita, Eduardo A. Undurraga, Jasmina Panovska-Griffiths

Table of contents

[Abbreviations 2](#_Toc103291324)

[Section A. Brief description of the previous studies used to extract the information for our modelling study 3](#_Toc103291325)

[Section B. Auxiliary analyses and key parameters computation 5](#_Toc103291326)

[Descriptive statistics of the data provided from the main study used for data extraction 5](#_Toc103291327)

[Calculating the $\varrho$ coefficient 6](#_Toc103291328)

[Estimating the $\rho$ parameter 7](#_Toc103291329)

[Estimating the $\beta h$ transmission coefficient 9](#_Toc103291330)

[Computing the rest of the parameters 11](#_Toc103291331)

[Section C. Model specifications and stability analysis 12](#_Toc103291332)

[Differential equations of the main model 12](#_Toc103291333)

[Basic reproduction number $R0$ 12](#_Toc103291334)

[Formula for the effective reproductive number over time (Rt): 14](#_Toc103291335)

[Section D. Additional figures 15](#_Toc103291336)

[Section E. Antibiotic- and bacteria-specific analyses for population dynamics 31](#_Toc103291337)

[Estimating the new antibiotic-specific $\rho$ parameter 33](#_Toc103291338)

[Estimating the new antibiotic-specific $\beta h$parameter 33](#_Toc103291339)

[New models for antibiotic specific GN-bacteria transmission dynamics 36](#_Toc103291340)

[New models for bacteria specific GN-ARB transmission dynamics 38](#_Toc103291341)

[Section F. Additional references 40](#_Toc103291342)

# Abbreviations

AMR: Antimicrobial resistant

ARB: Antibiotic resistant bacteria

ATB: Antibiotic

BSI: Bloodstream infections

ESCR: Extended-spectrum cephalosporin-resistant

FQ-R: Fluoroquinolone resistant

GN: Gram-negative

GN-ARB: Gram-negative antibiotic resistant bacteria

HICs: High income countries

LMICs: Low and middle income countries

MDR: Multidrug resistance

MDRGN: Multidrug resistant gram-negative bacteria

OECD: Organisation of Economic Cooperation and Development

WB: World Bank

WHO: World Health Organisation

# Section A. Brief description of the previous studies used to extract the information for our modelling study

We extracted the information on colonisation of antimicrobial-resistant bacteria in the community from a joint study comprising two research initiatives; the Maule Cohort (MAUCO) longitudinal study ^1^, and a parallel investigation to estimate the prevalence of multi-drug resistant GN bacteria (MDRGN) in the community ^2,3^.

First, the MAUCO cohort recruited 10,000 individuals (aged 38-74 years) from the region's total population (Molina). MAUCO's study aims to analyse the natural history of chronic diseases in the agricultural county of Molina ^1^.

Second, the MAUCO study participants' subsample was incorporated into an MDRGN posterior study from which faecal samples were obtained ^2,3^. Faecal samples were collected at participant's households (one per person) and then was sent to a lab to be stored at 4ºC for up to 24 hours before these samples were used for MDRGN screening purposed. Faecal samples were classified using the Antimicrobial Susceptibility Testing approach by implementing Tryptic Soy Agar (TSA) cultures based on MacConkey indicator to isolate gram-negative bacteria (two antibiotic types were tested: ceftazidime and ciprofloxacin). Finally, three types of bacteria-antibiotic combinations were analysed: fluoroquinolone-resistant (FQ-R), extended-spectrum cephalosporin-resistant (ESCR), and carbapenem-resistant. Resistant bacteria were coded as 1 for any gram-negative bacteria with intermediate resistance or resistant to at least one agent in three or more antimicrobial groups, including extended spectrum cephalosporins, fluoroquinolones, aminoglycosides, and carbapenems.

Epidemiological data included different self-reported characteristics divided into four domains: health system, environmental characteristics, sociodemographic and behavioural factors, and health status. First, the health system includes the number of hospital visits and antibiotic consumption during the previous six and three months (respectively). The number of times people visited the hospital's emergency department and the number of times people visited the hospital for medical checks. Secondly, environmental characteristics comprise whether people were exposed and the frequency in the number of contacts per week with animals such as dogs, cats, cows, pigs, ducks, chickens, and sheep. Another characteristic secondarily associated with this is whether the participant worked in the agricultural field being exposed to chemical contaminants. Thirdly, sociodemographic and behavioural factors included gender, age, education level, dietary intake, and food consumption. Fourth, health status encompasses the presence of any chronic condition (diabetes, cancer, cardiovascular and renal diseases, and high blood pressure). Table B1 describes a summary of the variables included in the analysis. Cardiovascular diseases and high blood pressure were excluded due to high missingness (>40%).

**Table A1** Sample characteristics and definitions from the main study used for data extraction

| **Individual-level characteristics** | |
| --- | --- |
| **Variables** | **Definition** |
| **Hospital level** | |
| hospitalization | Number of hospitalisations during the past six months |
| Antibiotic consumption | Dummy variable responding to the following question: Has the participant consumed antibiotics during the past three months? |
| **Environmental features** | |
| Animal contact | Did you have direct contact with “pigs, cows, ducks, chicken, turkey, sheep, horses, cat, dogs” during the last seven days?  *Direct contact is understood as whether the participant had contact with saliva, blood, urine, mucus, stools, or other animal body fluids. |
| Animal contact frequency | How many days did participants have contact with animals? |
| Agricultural occupation | Dummy variable indicating whether the participant work in the agricultural sector |
| Number of people in household | Continuous variable indicating the number of people living in household |
| **Sociodemographic and behavioural characteristics** | |
| Education | Educational level defined as three categories: These stand for 1: completed or uncompleted secondary education, 2: completed or uncompleted secondary high school, 3: more than high school. |
| Gender | Dummy variable indicating whether the participant is female or not |
| Age | Continuous variable indicating individual’s age in years |
| Food consumption (high fat red meat and dairy products) | Detailed dietary intake with frequencies by meal during the last seven days for dairy and high fat read meat. Categories stand for 0: never consumed in the last week, 1: one or less than one per week, 2: 2-4 times a week, and 3: 5-8 times a week. |
| **Health status** |  |
| Chronic diseases (diabetes and cancer) | dummy variables indicating whether the individual has been diagnosed with diabetes or cancer. |
| **Auxiliary variables:** Community level characteristics | |
| **Variables** | **Definition** |
| Annual quantity of births | Number of new-borns throughout 2018 in the county of Molina^1^ |
| Annual quantity of deaths | Number of deaths throughout 2018 in the county of Molina^1^ |

*Notes*: Community-level characteristics were obtained from publicly available sources. ^1^Figure B1 shows pictures of Molina.

# Section B. Auxiliary analyses and key parameters computation

We use the data from the existing studies to get some of the parameters for our modelling structure, and principally to get the environment risk coefficient. Firstly, we look at the data and describe it to compute a logistic model for the prevalence of GN-ARB to estimate our risk coefficient. These methods are detailed below, as well as the computation of the transmission and the rest of the critical parameters.

## Descriptive statistics of the data provided from the main study used for data extraction

Table B.1 shows the descriptive statistics in the Molina sample (N=357). Most participants were men (62.8%, SD=.48), 52.4 years of age (SD=9.53), and an uncompleted secondary education 0.78, SD=.64), (Table A1 and B1 for full variables details). The prevalence of ARB was 50.14% (95%CI=44.93;55.35). The average number of people living in a household was three, 16% of the sampled individuals consumed antibiotics during the last three months, and 15% people were hospitalized during the last six months (the same percentage reported working in agriculture). Participants tended to have contact with farming animals one day per week. These characteristics were adjusted to the entire population of Molina to compute the baseline conditions of our dynamic model. Therefore, Molina’s population stands for 46,000 inhabitants, so S(t0)= 23,064 while colonised population was 22,936. The latter was further divided between the infected population and hospitalised for further analyses.

**Table B1** Descriptive statistics of the sample (N=357)

| Variable | MEAN or % | SD | MIN | MAX |
| --- | --- | --- | --- | --- |
| Antibiotic resistance using faecal samples (%) | 50.14 | 0.50 | 0 | 1 |
| Number of people at household | 3.18 | 1.33 | 1 | 8 |
| Antibiotic consumption during last 3 months (%) | 15.96 | 0.37 | 0 | 1 |
| Contact w/dogs (days per week) | 3.65 | 3.42 | 0 | 7 |
| Contact w/cats (days per week) | 1.39 | 2.75 | 0 | 7 |
| Contact w/ducks (days per week) | 0.12 | 0.91 | 0 | 7 |
| Contact w/chicken (days per week) | 0.65 | 2.016 | 0 | 7 |
| High fat read meat consumption (categories) |  |  |  |  |
| None (%) | 39.04 |  | 0 | 1 |
| Less than once per week (%) | 39.89 |  | 0 | 1 |
| At least once per week (%) | 17.42 |  | 0 | 1 |
| Between 2 and 4 times per week (%) | 3.37 |  | 0 | 1 |
| Dairy products consumption (categories) |  |  |  |  |
| None (%) | 12.92 |  | 0 | 1 |
| Less than once per week or at once (%) | 39.33 |  | 0 | 1 |
| Between 2 and 4 times per week (%) | 35.88 |  | 0 | 1 |
| Between 5 and 8 times per week (%) | 11.58 |  | 0 | 1 |
| Agricultural occupation (%) | 14.16 | 0.35 | 0 | 1 |
| Hospitalised (%) | 14.77 | 0.47 | 0 | 1 |
| Diabetes (%) | 15.38 | 0.36 | 0 | 1 |
| Cancer (%) | 2.27 | 0.15 | 0 | 1 |
| Age in years | 52.38 | 9.53 | 37 | 74 |
| Education level (categories) |  |  |  |  |
| Completed/uncompleted primary education (%) | 33.61 |  | 0 | 1 |
| Completed/uncompleted secondary education (%) | 54.62 |  | 0 | 1 |
| More than secondary education (%) | 11.76 |  | 0 | 1 |
| Gender (1: Male) | 62.75 | 0.48 | 0 | 1 |

*Notes:* ^2^ categories stand for 0: never consumed in the last week, 1: one or less than one per week, 2: 2-4 times a week, and 3: 5-8 times a week.

## Calculating the $\varrho$ coefficient

The coefficient indicating exposure to ARB reservoirs was formed as the average of selected variables being associated with higher ARB levels. The variables included were people who lived with more than four people at home (17.23%), people visiting the hospital during the latest six months (5.1%), proportion of people who had direct contact with animals in the last week (11.86%) (pig, cow, sheep, ducks, horse, and chicken), people who worked on the agricultural field (27.2%), people taking antibiotics during the last 6 months (15.96%), and the proportion of people having a dietary intake above the median consumption in terms of meat, fish, bacon, white meat, and dairy products (cheese, milk, yogurt, etc); 30,98%.

## Estimating the $\rho$ parameter

The $\rho$ parameter captures the risk for individuals in acquiring resistant bacteria in the community based on different exposures. To compute the parameter, we employed a three stages analysis. First, a logistic regression using robust standard errors was used to estimate the adjusted prevalence of ARB (Equation C2 and Table C3). Independent variables used are detailed in Equation C2; auxiliary variables such as age, underlying health conditions, and sex were also added to isolate the main association. Table C3 shows that an increase of one person living in a household is associated with a 19% increase in the ARB prevalence (OR= 1.19, SE=.13). Antibiotic consumption positively associated with ARB prevalence (OR=2.02, SE=.72). Therefore, we predicted the regression model results to obtain the adjusted prevalence of ARB (Figure C4 for normality checks). Secondly, we created a new dummy variable ($\hat{\psi}$) taking the value of 1 for predicted ARB values $\geq$0.5, and 0 for predicted ARB values $\leq$0.5. Thirdly, ($\hat{\psi}$) was cross-tabulated with the former proportion of exposure to potential ARB reservoirs ($\varrho$). Table C5 shows the results of the comparison table between both variables ($\varrho$ and $\hat{\psi}$) from which the final risk ratio ($\rho$) was calculated. The risk ratio was 1.27 times higher for exposed individuals to acquire resistant bacteria compared with partly unexposed individuals.

**Equation B2** Logistic model structure

$$\log\left( Resistance \right)=\beta_{0}+ \beta_{1}{Number of people at household}_{i}+ \beta_{2}{Antibiotic consumption}_{i}+ \beta_{k}{Contact with animals}_{i}+ \beta_{7}{Highfat read meat consumption}_{i}+ \beta_{8}{Dairy products consumption}_{i}+ \beta_{9}{Agricultural occupation}_{i}+ \beta_{10}{Previous hospitalization}_{i}+ \beta_{j}X_{im}+ \mu_{i} ;$$

$\forall individuo i.$ “k” ranges between 3 and 6. “j” ranges between 11 and 14. “m” stands for $m características socio-demográficas$, including chronic conditions (cancer and diabetes), age in years, and education level.

**Table B3** Logistic regression results (N=357)

| Probability of being bacterial resistant | OR | SE | P-value |
| --- | --- | --- | --- |
| Number of people at household | 1.19* | 0.13 | 0.100 |
| Antibiotic consumption during last week | 2.02** | 0.72 | 0.047 |
| Contact w/dogs (days) | 0.95 | 0.03 | 0.164 |
| Contact w/cats (days) | 0.97 | 0.05 | 0.498 |
| Contact w/ducks (days) | 1.08 | 0.13 | 0.489 |
| Contact w/chicken (days) | 0.94 | 0.06 | 0.331 |
| High fat read meat consumption ^(a)^ | 1.07 | 0.17 | 0.684 |
| Dairy products consumption ^(a)^ | 1.16 | 0.17 | 0.297 |
| Agricultural occupation | 1.05 | 0.41 | 0.894 |
| Hospitalised | 0.85 | 0.23 | 0.539 |
| Diabetes | 1.38 | 0.17 | 0.010 |
| Cancer | 1.68 | 0.37 | 0.018 |
| Age in years | 1.02 | 0.01 | 0.244 |
| Education level ^(a)^ | 0.84 | 0.17 | 0.397 |
| Constant | 0.05** | 0.06 | 0.014 |

*Notes*: * p<0.1, ** p<0.05, *** p<0.01, robust standard errors were used. OR stands for Odds Ratio. ^(a)^ These variables were comprised into a single category.


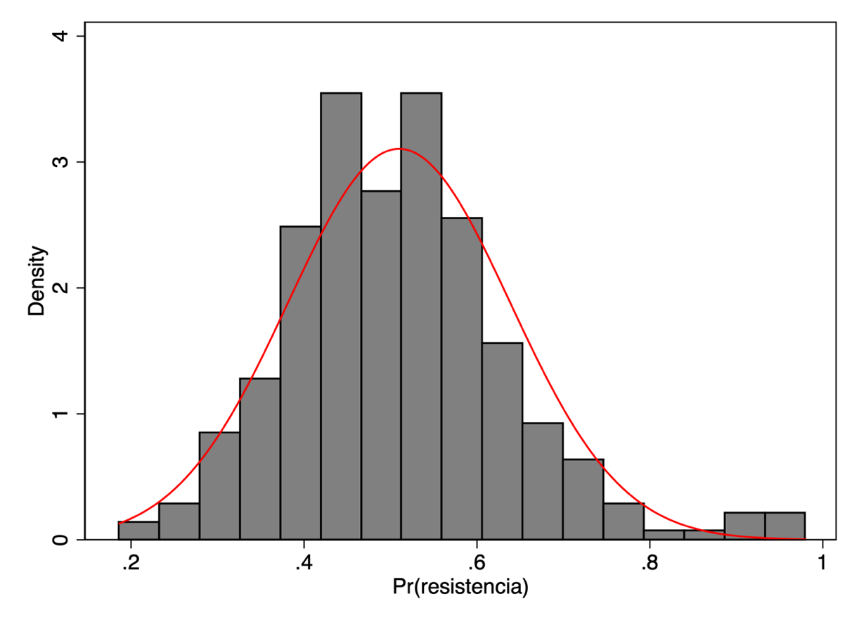


**Figure B4 Histogram of the predicted values from Table B.1.** The red line indicates a normal distribution.

**Table B5** Comparison table for exposed and unexposed groups (values indicate the number of individuals)

$$\hat{\psi}$$

$$\varrho$$

|  | Resistant bacteria | Susceptible bacteria | $\Sigma$ | Risk |
| --- | --- | --- | --- | --- |
| Exposed (E) | 121 | 64 | 185 | 65.4% |
| Unexposed (U) | 88 | 84 | 172 | 51.16% |
| $\Sigma$ | 209 | 148 | 357 |  |

*Notes*: Exposed and unexposed groups were based on the probabilities shown in the main model (Figure 2). Resistant bacteria prevalence was calculated based on the predicted values from Table B.2 whereas probabilities above .5 were catalogued as resistant, and below this value were classified as susceptible.

*Risk ratio 🡪 65.4/51.16 = 1.27*

*Odd ratio 🡪 121*84 /88*64 = 1.81*

- *Interpretation: Both indicators show an increased risk/odd of resistance for the exposed group.*

## Estimating the $\beta_{h}$ transmission coefficient

$\beta_{h}$ was computed based on previous empirical studies ^4,5^. We used the average number of individual cases colonized by GN-ARB from 2009 to 2017 in four main Chilean southern regional hospitals. This includes the regional hospitals in *O’Higgins, Biobio, Araucania,* and *Maule*. A general model was computed by using a function [f(x)] adjusted to our modelling parameters, which depended on the main structure of our modelling. Table C6 displays that $\beta_{h}$=0.0005308, and it had the best fit ($R^{2}$=.87), compared to other traditional functional forms (exponential, polynomial, Gaussian). Figure C7 depicts how well the function was adjusted to the observed data points with its respective 95% CI added. Finally, we multiplied the $\beta_{h}$ by 0.25 as previous literature has suggested for the equivalence between transmission rates between the hospital and community ^6^.

**Table B6** Results of the adjusted function (curve fitting) to calculate $\beta$_h_

| Coefficient (95% CI):$\beta$_h_ = 0.000525 (0.00047, 0.000578) |
| --- |
| Goodness of fit: |
| SSE: 787.6 |
| $R^{2}$: 0.8725 |
| Adjusted $R^{2}$: 0.8725 |
| RMSE: 426.6 |

*Notes*: General model adjusted by a function f(x)= (beta, x). Least absolute residual robust errors were computed. 0.00001 was used a start-point. ^1^see the code in Appendix, section D.


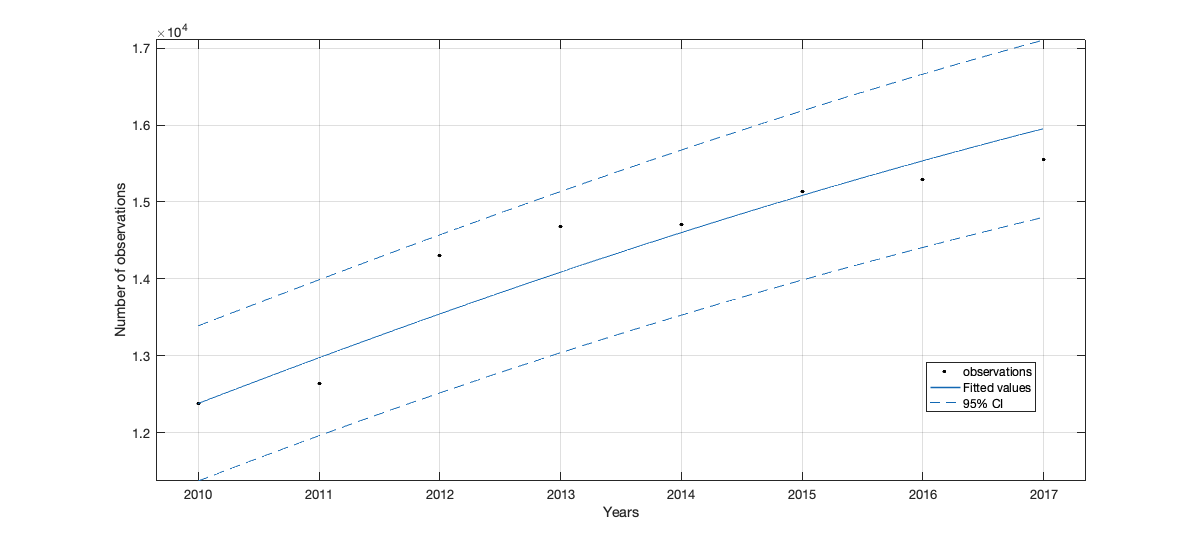


x 10^4^ x 2

**Figure B7 Curve fitting output to estimate beta (**$\boldsymbol{\beta}_{\boldsymbol{h}}\boldsymbol{)}$**.** The fitting curve tool on MATLAB was used to estimate the transmission parameter based on the model specifications. Data used consisted of an average of the prevalence of three GN-ARB reported before ^7^. These bacteria are *Pseudomonas aeruginosa, Klebsiella pneumoniae, Acinetobacter* *baumanii*, and *Escherichia* *coli*. Bacteria were resistant to either carbapenems, quinolones, cephalosporins, or aminoglycosides. We used data from 6 public hospitals in the highlighted regions (Araucanía, Biobio, Maule, and O’Higgins). The average prevalences were 26.91%, 27.48%, 31.08%, 31.93%, 31.98%, 32.89%, 33.25% and 33.80% over an eight years period (2010 through 2017). We adjusted those prevalences to our population sizes.

## Computing the rest of the parameters

- Turnover of the hospital population in Molina.

[907 annual individuals hospitalized in 2018 / population size=45,976]/ 365 days), according to the Chilean Ministry of Health ([DEIS](https://deis.minsal.cl/)). Therefore, the turnover is 0.000054 per day.

- Calculation of the number of people colonised by GN-ARB in the hospital (H)

| Total annual hospital discharges in 2017 | 75,091 |
| --- | --- |
| Population size in Molina (number of people) | 46,000 |
| Daily discharges 2017 (annual discharges/365) | 205.728767 |
| Ratio (population size/daily discharges) | 0.00447236 |
| Prevalence of GN-ARB in the hospital (%) ^a^ | 57% |
| Parameter (ratio*prevalence) | 0.00254925 |
| H= | 0.0025*N |
| Notes: Annual hospital discharges data obtained from the Chilean Department of Statistics and Health Information ([DEIS](https://informesdeis.minsal.cl/SASVisualAnalytics/?reportUri=%2Freports%2Freports%2F23138671-c0be-479a-8e9d-52850e584251&sectionIndex=0)). ^a^The prevalence of GN-ARB was extracted from a previous study carried out in Chile in the regional hospital of Curico (southern area) ^3^. N stands for the population size. | |

# Section C. Model specifications and stability analysis

For this mathematical model, there is a domain ($\mathbb{C)}$ where the system of equations is defined from a mathematical and epidemiological perspective.

$\mathbb{C}$ : = {(S, Z_c_, Z_h_, I_c_, I_hh_, I_hc_, R_c_, R_h_) ∈ ($R_{0}^{+}$)8}

The domain $\mathbb{C}$ is valid epidemiologically as populations are all non-negative. After constraining the derivatives of our compartments from Equation D.1 (see below) to nought:

$\frac{dS}{dt},\frac{dZc}{dt},\frac{dZh}{dt},\frac{dIc}{dt},\frac{dIhh}{dt}$, $\frac{dIhc}{dt}$ $,\frac{dRc}{dt}$ $,\frac{dRh}{dt}$ = 0

We can get our solutions from a point where there is no variation in the results over time. Hence, all compartments are equal to naught. We linearized the equations about the disease-free equilibrium (using the solutions from above). We computed the eigenvalues of the corresponding Jacobian matrix (which shows first-order partial derivatives of our compartmental model).

## Differential equations of the main model

$\frac{dS}{dt} =\Lambda N+\gamma Z_{C}+\gamma Z_{H}-\rho\epsilon\left( \beta_{C}SZ_{C} \right)-\rho\delta\epsilon(\beta_{H}SZ_{H})-\phi S$ (1)

$\frac{dZ_{C}}{dt}=\rho\epsilon\left( \beta_{C}SZ_{C} \right)+\omega_{C}I_{C}+\alpha Z_{H}-\xi_{C}\left( 1-\gamma\right){\left( 1-\delta\right)Z}_{C}-\delta Z_{C} -\gamma Z_{c}-\phi Z_{C}$ (2)

$\frac{dZ_{H}}{dt}=\rho\delta\epsilon\left( \beta_{H}SZ_{C} \right)+\delta Z_{C}+\omega_{H}\left( I_{HH}+I_{HC} \right)-\xi_{H}\left( 1-\alpha\right)Z_{H}-{\alpha Z}_{H}-\gamma Z_{H}-\phi Z_{H}$ (3)

$\frac{dI_{C}}{dt}=\xi_{C}\left( 1-\gamma\right){(1-\delta)Z}_{C}-\delta_{I}I_{C}-\omega_{C}I_{C}-(1-\omega_{c})(1-\delta_{I}){\eta V}_{C}I_{C}-\phi I_{C}$ (4)

$\frac{dI_{HC}}{dt}= \delta_{I}I_{C}-\omega_{H}I_{HC}-\left( 1-\omega_{H} \right)\eta V_{H}I_{HC}-\phi I_{HC}$ (5)

$\frac{dI_{HH}}{dt}= \xi_{H}\left( 1-\alpha\right)Z_{H}-\omega_{H}I_{HH}-{\left( 1-\omega_{H} \right)\eta V}_{H}I_{HH}-\phi I_{HH}$ (6)

$\frac{dR_{C}}{dt}=\left( 1-\omega_{C} \right)(1-\delta_{I})\eta V_{C}I_{C}$ (7)

$\frac{dR_{H}}{dt}=\left( 1-\omega_{H} \right)\eta V_{H}I_{H}$ (8)

***Notes****:* *subscript “r” stands for resistance branch while “s” for susceptible branch. Equations are represented in Figure 1.*

## Basic reproduction number $\boldsymbol{R}_{\mathbf{0}}$

The basic reproduction number was calculated using the “Next Generation Matrix Approach” presented in (Heffernan, 2005 & Van den Driessche, 2017) ^8,9^. Using equation (1), we describe the $F$ and $V$ as:

$$F=\left[ \begin{matrix} \rho\epsilon\beta_{C}S_{0} & 0 & 0 & 0 \\ 0 & \rho\delta{\epsilon\beta}_{H}S_{0} & 0 & 0 \\ 0 & 0 & 0 & 0 \\ 0 & 0 & 0 & 0 \end{matrix} \right]$$

$$V=\left[ \begin{matrix} \xi_{C}\left( 1-\gamma\right)\left( 1-\delta\right)+\delta+\gamma+\phi& -\alpha& -\omega_{C} & 0 \\ -\delta& \xi_{H}\left( 1-\alpha\right)+\alpha+\gamma+\phi& 0 & {-\omega}_{H} \\ -\xi_{C}(1-\gamma)(1-\delta) & 0 & \delta_{I}+\omega_{C}+\left( 1-\omega_{C} \right)\left( 1-\delta_{I} \right)\eta V_{C}+\phi& 0 \\ 0 & -\xi_{H}(1-\alpha) & {-\delta}_{I} & \omega_{H}+\left( 1-\omega_{H} \right)\eta V_{H}+\phi\end{matrix} \right]$$

Where;

$$F=\left[ \frac{\partial\mathcal{F}_{i}(x_{0})}{\partial x_{j}} \right];V=\left[ \frac{\partial\mathcal{V}_{i}(x_{0})}{\partial x_{j}} \right]$$

here $\mathcal{F}_{i}(x)$ is the rate of appearance of new infections in infected compartment $i$, $\mathcal{V}_{i}(x_{0})$ is the rate of other transitions between compartment $i$ and other compartments and $x_{0}\in R^{n}$ is the initial condition.

Then the next generation matrix $FV^{-1}$ was calculation using the parameters and initial conditions of the system. The results are:

$$Low Risk Escenario$$

$$FV^{-1}= \left[ \begin{matrix} 1.13171696 & 1.04176171 & -8.06958317x{10}^{-1} & 9.36447503x{10}^{-1} \\ -2.47883017x{10}^{-8} & 2.33270055x{10}^{-5} & -1.95753580x{10}^{.-5} & 2.09688221x{10}^{-5} \\ 0 & 0 & 0 & 0 \\ 0 & 0 & 0 & 0 \end{matrix} \right]$$

$$High Risk Escenario$$

$$FV^{-1}= \left[ \begin{matrix} 1.46268054 & 1.32303737 & -1.02483706 & 1.18928833 \\ -2.47883017x{10}^{-8} & 2.33270055x{10}^{-5} & -1.95753580x{10}^{.-5} & 2.09688221x{10}^{-5} \\ 0 & 0 & 0 & 0 \\ 0 & 0 & 0 & 0 \end{matrix} \right]$$

Then we calculate the eigenvalues of the next generation matrix:

And the basic reproduction number is the greatest eigenvalue of $FV^{-1}$.

$$Low Risk Escenario R_{0}=1.151717$$

$$High Risk Escenario R_{0}=1.462681$$

$R_{0}$>1 means that the disease is permanent in the community and will persist in the future, so the disease will not die out completely. This is in line with the literature and the WHO ^10,11^. Even though vital dynamics are included in the model, raising the number of susceptible individuals over time, the spread of resistant organisms is equivalent to 1 person passing on the virus to another one. Also, the proportion of people already colonised in the community at baseline is sharp.

Following the same approach, the parameter of low exposure to antibiotic reservoirs ($\rho$=1.0), give us an $R_{0}=$0.8198.

## Formula for the effective reproductive number over time (Rt):

R_t_ = R_0_ * (S_t_/N_t_) ;

S_t_= susceptible population at time ‘t’, while N_t_= total population at time ‘t’

**Figure C1.** Calculated effective reproductive number R_t_ over time since t=1 and by $\rho$ coefficient

# Section D. Additional figures

**Figure D1 Population size over time, by risk coefficient (low and high exposure to ATB reservoirs).** Estimates obtained from Table 1, Equations

**Figure D2** **Deaths attributed to GN-ARB bloodstream infections in the community (non-hospitalised patients), by risk coefficient (low and high exposure to ATB reservoirs)*.*** Estimates obtained from Table 1, Equations

**Figure D3 Deaths attributed to GN-ARB bloodstream infections in hospitalised patients, by risk coefficient (low and high exposure to ATB reservoirs).**  Estimates obtained from Table 1, Equations


**Figure D4.1** Population dynamics using $\rho$=1.0 and a Monte Carlo simulation for the beta parameter. Estimations based on initial conditions stated in Table 1, model scheme in Figure 2.


**Figure D4.2** **Population dynamics using** $\boldsymbol{\rho}$**=1.27 and a Monte Carlo simulation for the beta parameter** Estimations based on initial conditions stated in Table 1, model scheme in Figure 2.

**(A)**

**(B)**

**(C)**

**(D)**

**Figure D5.1. Population dynamics for the main compartments using a low and high-risk exposure to ATB reservoirs (**$\boldsymbol{\rho}\boldsymbol{=}$ **1 and 1.27, respectively).** S stands for susceptible, Z_C_ for colonised by a GN-ARB. I_C_ for infected individuals by a GN-ARB, I_HH_ for infected individuals at the hospital with hospital-acquired infection. I_HC_ is for individuals infected by a GN-ARB at the hospital but with community-acquired infection. Risk coefficient=1.27 means higher exposure to GN-ARB reservoirs while $\rho$=1 means lower exposure. Full results of the complete system can be found in supplementary material Figures D1-D4.


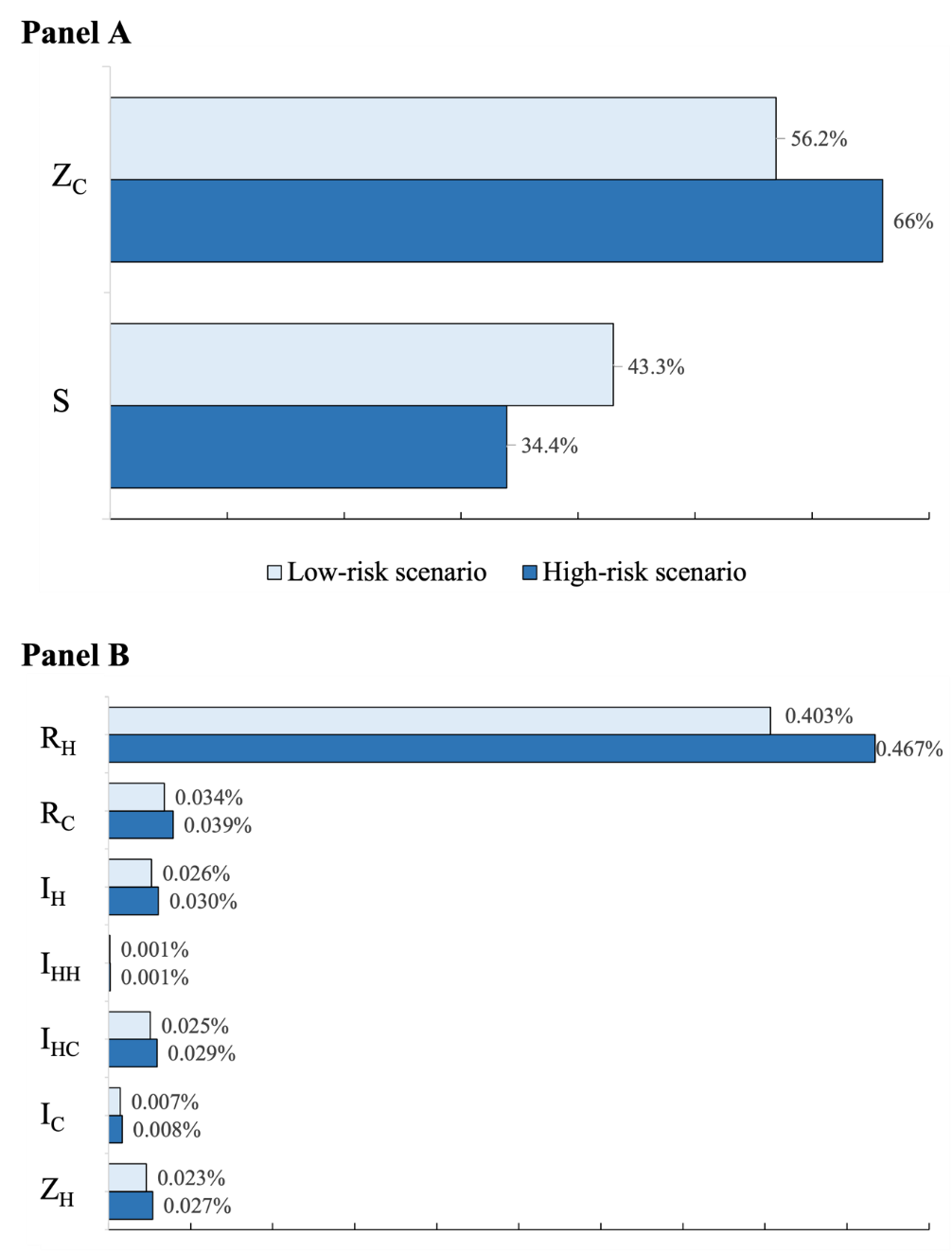


**Figure D5.2 Proportion of the population (N) per compartment and by risk-scenario at the end of the estimated period.**  All groups coloured in light blue (or dark) sum up 100% of the population (N) including those removed. The proportions were calculated from the primary model over the end of the study period (also see Figure D5, Supplementary Material). S: Susceptible population, Z_C_: Colonised individuals by a GN-ARB in the community; R_C_: Individuals removed (dead due to GN-ARB BSI in the community); R_H_: Individuals removed (dead due to GN-ARB BSI in the hospital); I_H_: Individuals with a GN-ARB BSI in the hospital; I_HH_: Individuals with a hospital-acquired GN-ARB BSI; I_HC_: Individuals with a community-acquired GN-ARB BSI; I_H_: Individuals with a GN-ARB BSI in the community; Z_H_: Colonised individuals by a GN-ARB in the hospital.

**Figure D6.1 Univariate analysis for the impact of variation in the hospitalisation rate due to GN-ARB BSI levels on the number of people infected and the total attributed deaths, using** $\boldsymbol{\rho}$**=1.0 (low-risk scenario of exposure to ATB reservoirs).** Bottom-left panne figure uses the same values for the sigma parameter as the other; however, only extreme values. Are shown due to the narrow space between lines.

**Figure D6.2 Univariate analysis for the impact of variation in the hospitalisation rate due to GN-ARB BSI levels on the number of people infected and the total attributed deaths, using** $\boldsymbol{\rho}$**=1.27 (high-risk scenario of exposure to ATB reservoirs).** Bottom-left panne figure uses the same values for the sigma parameter as the other figures; however, only extreme values are shown due to the narrow space between lines. Estimates were obtained from the main model (Table 1-2, Figure 2 main manuscript).

**Figure D7.1** **Univariate sensitivity analysis for the impact of the probability of a bacterial resistant bloodstream infection to occur in the community on the number of people infected and attributed deaths in the community and hospital settings in the low-risk scenario (ρ=1.0, low-risk exposure to ATB reservoirs).** Estimates obtained from the main model (Table 1-2, Figure 2 main manuscript). Estimates were obtained from the main model (Table 1-2, Figure 2 main manuscript).

**Figure D7.2 Univariate sensitivity analysis for the impact of the Probability of a bacterial resistant bloodstream infection to occur in the community on the number of people infected and attributed deaths in the community and hospital settings in the high-risk scenario (ρ=1.27, high-risk exposure to ATB reservoirs**). Estimates obtained from the main model (Table 1-2, Figure 2 main manuscript). Estimates were obtained from the main model (Table 1-2, Figure 2 main manuscript).

**(a)**

**(b)**

**(a)**

**Figure D8 Univariate analysis for the probability of acquiring a GN-ARB BSI and its impact on the number of people infected within the hospital setting by risk coefficient.** (a) $\rho$ =1.0 (low-risk exposure to ATB reservoirs), (b) $\rho$ =1.27 (high-risk exposure to ATB reservoirs). Estimates obtained from the main model (Table 1-2, Figure 2 main manuscript).

**(a)**

**(b)**

 **Figure D9** **Univariate analysis for the treatment in the hospital parameter and its impact on the number of people infected in the hospital, by risk coefficient.** (a) $\rho$ =1.00 (low-risk exposure to ATB reservoirs), (b) $\rho$ =1.27 (high-risk exposure to ATB reservoirs). Bottom-left panel figures from (a) and (b) uses the same values W_h_ parameter as the other figures, however, only extreme values are shown due to the narrow space between lines. Estimates obtained from the main model (Table 1-2, Figure 2 main manuscript).

**(a)**

**(b)**


**Figure D**.**10** **Univariate analysis of the impact of changes in bacterial spontaneous clearance of colonization γ on the number of people infected by GN-ARB in the community and hospital setting, by risk coefficient.** (a) $\rho$ =1.0 (low-risk exposure to ATB reservoirs), (b) $\rho$ =1.27 (high-risk exposure to ATB reservoirs).

**(a)**

**(b)**

**Figure D.11 Univariate analysis for the number of people within each compartment by analysing different transmission levels and by risk coefficient.** (a) $\rho$ =1.0 (low-risk exposure to ATB reservoirs), (b) $\rho$ =1.27 (high-risk exposure to ATB reservoirs). Notes: Estimates obtained from the main model (Table 1-2, Figure 2 main manuscript).

# Section E. Antibiotic- and bacteria-specific analyses for population dynamics

**Table E1.** Descriptive statistics of antibiotic-specific resistance rates among individuals carrying GN-bacteria in the community (N=357)

| Variable | MEAN (%) | SD |
| --- | --- | --- |
| Antibiotic resistance {quinolones} | 39.49 | 0.50 |
| Antibiotic resistance {cephalosporins} | 28.85 | 1.33 |
| Antibiotic resistance {carbapenems} | 5.60 | 0.37 |

Notes: SD stands for standard deviation

**Table E2.** Logistic regression results of antibiotic-specific resistance rates among individuals carrying GN-bacteria in the community (N=357)

|  | Resistance to quinolones | | Resistance to cephalosporins | | Resistance to carbapenems | |
| --- | --- | --- | --- | --- | --- | --- |
| Probability of being bacterial resistant | OR | SE | OR | SE | OR | SE |
| Number of people at household | 1.188* | -0.12 | 1.059 | -0.12 | 0.834 | -0.23 |
| Antibiotic consumption during last week | 2.011** | -0.67 | 2.252** | -0.77 | 1.322 | -0.93 |
| Contact w/dogs (days) | 0.955 | -0.04 | 1.02 | -0.04 | 0.895 | -0.08 |
| Contact w/cats (days) | 0.989 | -0.05 | 0.962 | -0.06 | 0.858 | -0.15 |
| Contact w/ducks (days) | 1.149 | -0.16 | 0.936 | -0.16 | 1.00 | 1.00 |
| Contact w/chicken (days) | 0.945 | -0.07 | 0.991 | -0.08 | 1.085 | -0.19 |
| High fat read meat consumption ^(a)^ | 1.072 | -0.17 | 1.075 | -0.18 | 0.61 | -0.25 |
| Dairy products consumption ^(a)^ | 0.993 | -0.15 | 1.408** | -0.23 | 1.491 | -0.54 |
| Agricultural occupation | 0.884 | -0.34 | 1.166 | -0.48 | 1.00 | 1.00 |
| Hospitalised | 0.87 | -0.24 | 0.992 | -0.29 | 1.264 | -0.78 |
| Diabetes | 1.12 | -0.4 | 0.918 | -0.35 | 0.458 | -0.34 |
| Cancer | 1.379 | -1.21 | 2.762 | -3.15 |  |  |
| Age in years | 1.008 | -0.01 | 1.026 | -0.02 | 0.996 | -0.03 |
| Education level (Ref.: Completed/uncompleted primary education) | | | | | | |
| Completed/uncompleted secondary education (%) | 0.600* | -0.17 | 1.064 | -0.33 | 2.306 | -1.73 |
| More than secondary education (%) | 0.81 | -0.34 | 0.908 | -0.44 | 0.791 | -0.99 |
| Constant | 0.163 | -0.36 | 0.006* | -0.02 | 0.321 | -0.94 |

*Notes*: * p<0.1, ** p<0.05, *** p<0.01, robust standard errors (SE) were used. OR stands for Odds Ratio. ^(a)^ These variables were comprised into a single category.

**
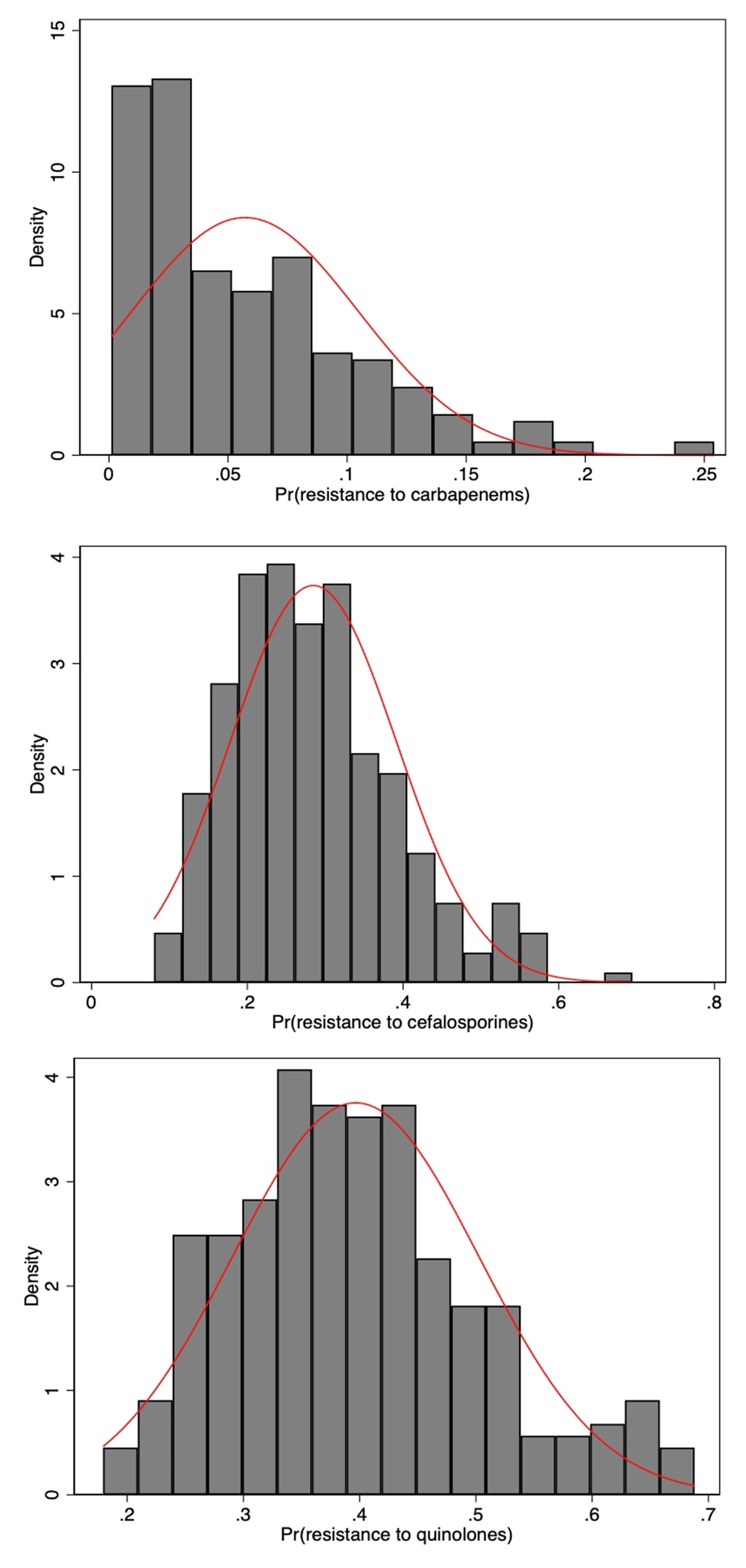
**

**Figure E1. Distribution of each antibiotic-specific resistance rate.** Red line indicates a normal distribution

## Estimating the new antibiotic-specific $\rho$ parameter

**Table E3.** Comparison table for exposed and unexposed groups for quinolone, cephalosporins, and carbapenem resistant GN bacteria (values indicate the number of individuals)

|  | Risks for quinolone resistant GN bacteria | Risk for cephalosporins resistant GN bacteria | Risk for carbapenems resistant GN bacteria |
| --- | --- | --- | --- |
| Exposed (E)  $\varrho$ | 56% | 50% | 41% |
| Unexposed (U) | 36% | 39% | 40% |
| $RR$ {$\rho$ parameter} | 1.57 | 1.30 | 1.01 |

*Notes*: Exposed and unexposed groups were based on the probabilities showed in the main model (Figure 2). Resistant bacteria prevalence was calculated based on the predicted values from of Table E2, probabilities above the average predicted values were catalogued as resistant, and below this value were classified as susceptible.

## Estimating the new antibiotic-specific $\beta_{h}$parameter


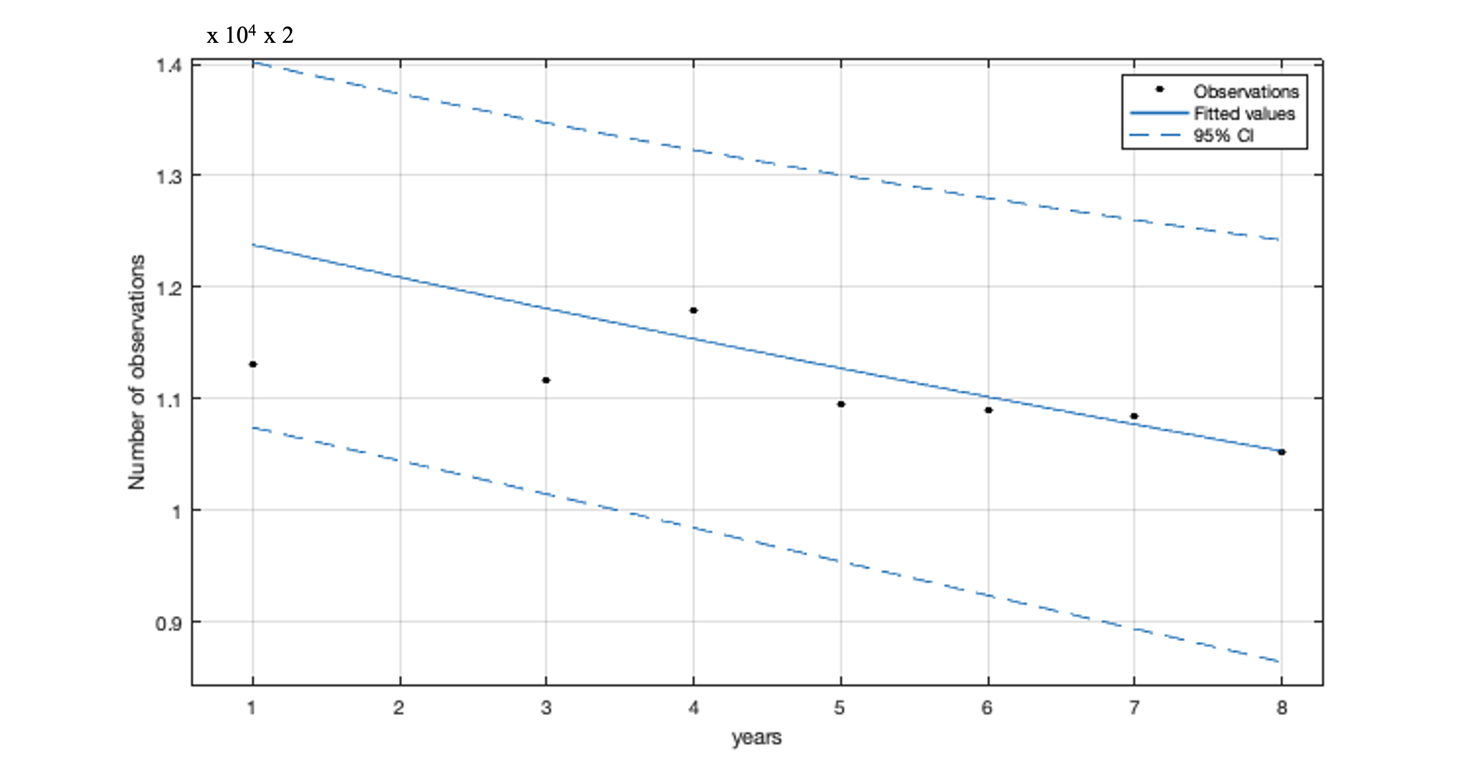


**Figure E2 Curve fitting output to estimate the beta (**$\boldsymbol{\beta}_{\boldsymbol{h}}\boldsymbol{)}$ **parameter for quinolone resistant GN bacteria.** The fitting curve tool on MATLAB was used to estimate the transmission parameter based on the model specifications. Data used consisted of an average of the prevalence of three GN-ARB reported before ^7^. These bacteria are *Pseudomonas aeruginosa, Klebsiella pneumoniae, Acinetobacter* *baumanii*, and *Escherichia* *coli*. Bacteria were resistant to either carbapenems, quinolones, cephalosporins or aminoglycosides. We used data from 6 public hospitals located in the highlighted regions (Araucanía, Biobio, Maule and O’Higgins), over an eight years’ time period (from 2010 to 2017). We adjusted those prevalences to our population sizes.


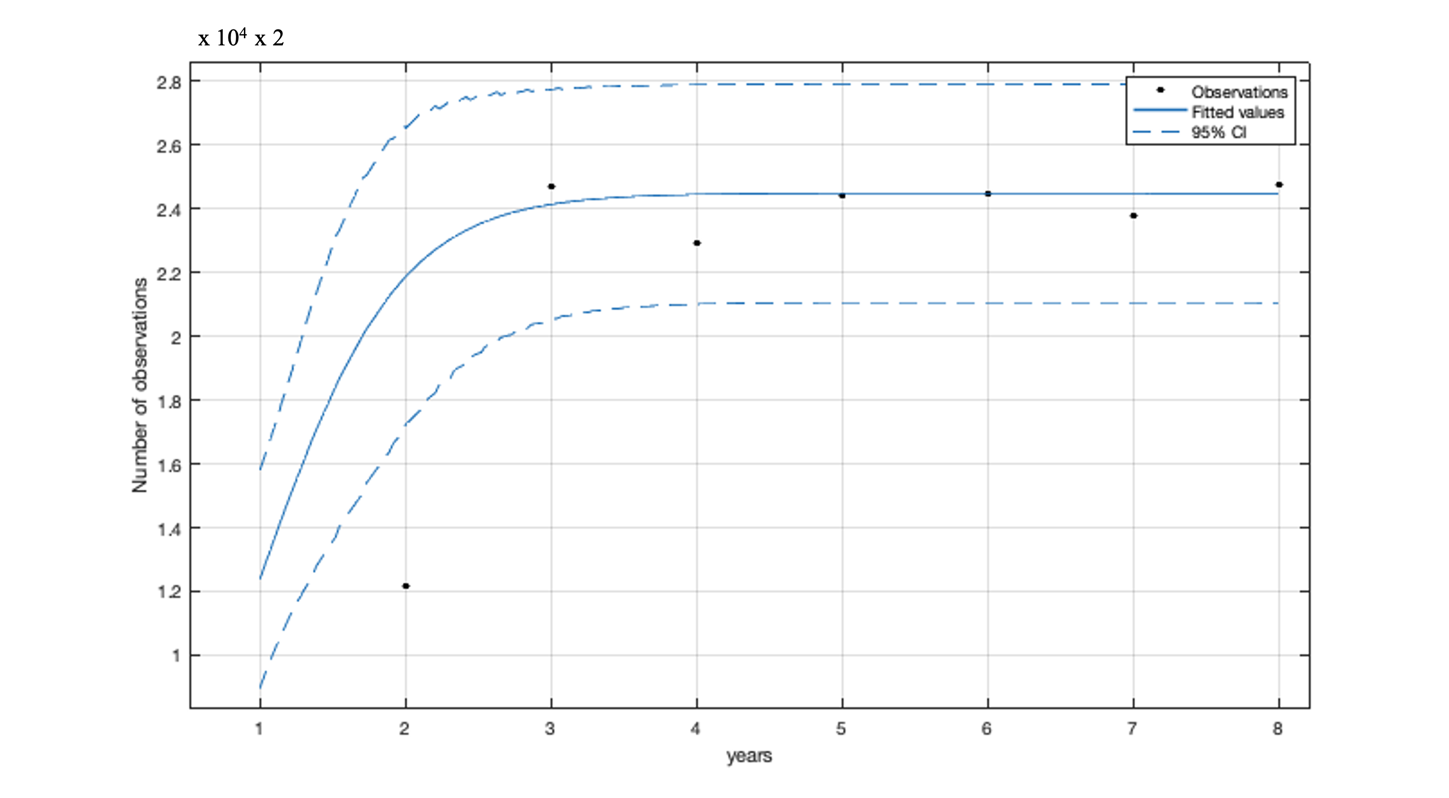


**Figure E3 Curve fitting output to estimate the beta (**$\boldsymbol{\beta}_{\boldsymbol{h}}\boldsymbol{)}$ **parameter for cephalosporins resistant GN bacteria**


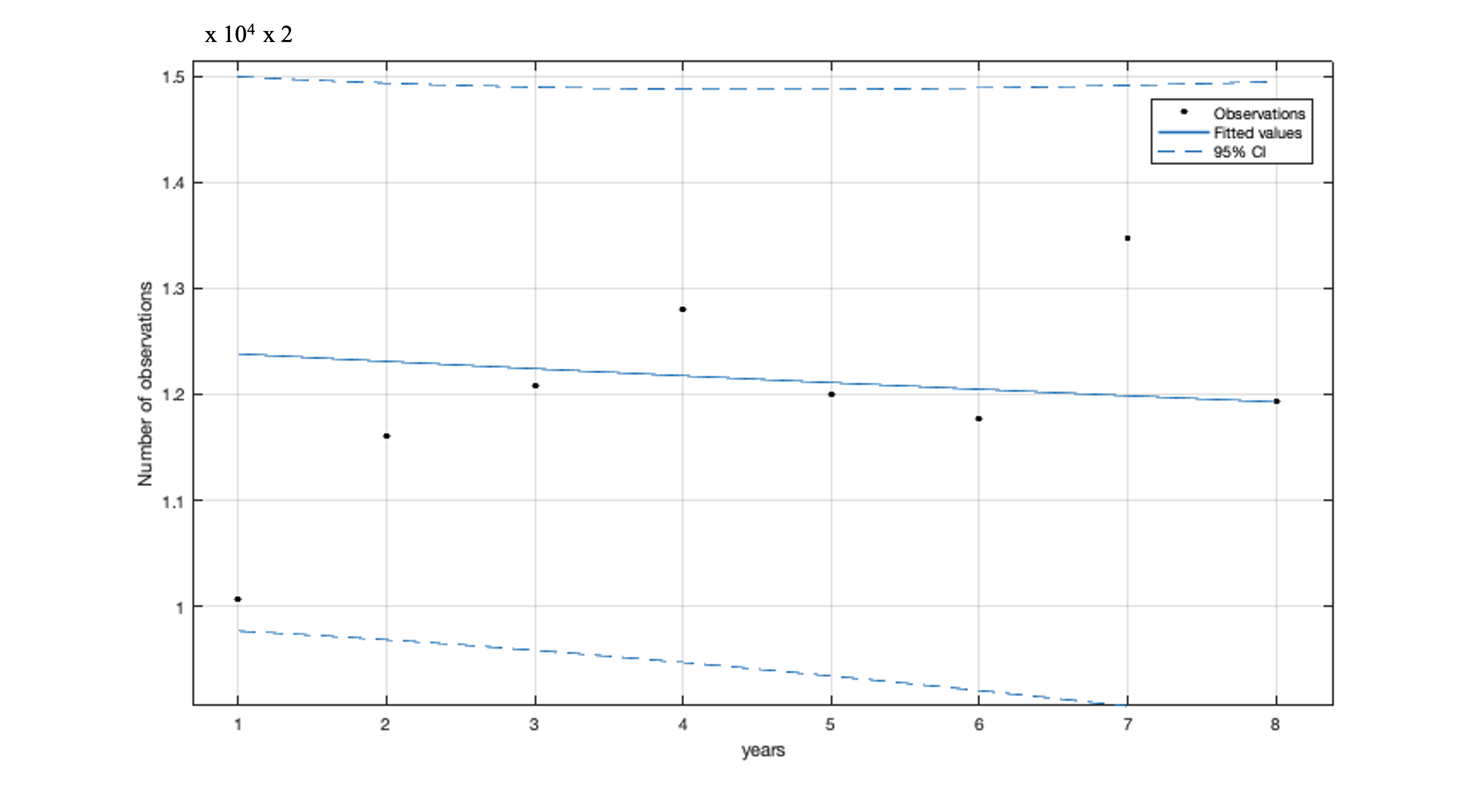


**Figure E4 Curve fitting output to estimate the beta (**$\boldsymbol{\beta}_{\boldsymbol{h}}\boldsymbol{)}$ **parameter for carbapenem resistant GN bacteria.** The fitting curve tool on MATLAB was used to estimate the transmission parameter based on the model specifications. Data used consisted of an average of the prevalence of three GN-ARB reported before ^7^. These bacteria are *Pseudomonas aeruginosa, Klebsiella pneumoniae, Acinetobacter* *baumanii*, and *Escherichia* *coli*. Bacteria were resistant to either carbapenems, quinolones, cephalosporins or aminoglycosides. We used data from 6 public hospitals located in the highlighted regions (Araucanía, Biobio, Maule and O’Higgins), over an eight years’ time period (from 2010 to 2017). We adjusted those prevalences to our population sizes.

**Table E4** Results of the adjusted function (curve fitting) to calculate each antibiotic-specific $\beta$_h_ parameter

| **Quinolones-resistant GN bacteria** |
| --- |
| Coefficient (95% CI):$\beta$_h_ = 0.00006171 (-1.656e-05, 0.00014) |
| Goodness of fit: |
| SSE: 3.353e+06 |
| $R^{2}$: 0.8092 |
| Adjusted $R^{2}$: 0.8092 |
| RMSE: 692.1 |
| \| **Cephalosporins-resistant GN bacteria** \| \| --- \| \| Coefficient (95% CI):$\beta$_h_ = 0.006831 (0.002638, 0.01102) \| \| Goodness of fit: \| \| SSE: 1.459e+07 \| \| $R^{2}$: 0.9589 \| \| Adjusted $R^{2}$: 0.9589 \| \| RMSE: 1444 \| \| **Carbapenems-resistant GN bacteria** \| \| Coefficient (95% CI):$\beta$_h_ = 0.0001766 (5.222e-05, 0.0003009) \| \| Goodness of fit: \| \| SSE: 8.542e+06 \| \| $R^{2}$: 0.267 \| \| Adjusted $R^{2}$: 0.267 \| \| RMSE: 1105 \| |

*Notes*: General model adjusted by a function of the form f(x)= (beta, x). Least absolute residual robust errors were computed. 0.00001 was used a start-point.

## New models for antibiotic specific GN-bacteria transmission dynamics

We varied the transmission parameter in hospitals (β_H_), the risk parameter (ρ) and the initials conditions according to three different risk scenarios regarding antibiotic-specific resistance for quinolones, cephalosporines and carbapenems. We simulated the evolution of the different compartment overtime (see the parameters used below). Consecutively, we compared the simulations obtained against our baseline model (GN-ARB transmission) for the low- and high-risk scenario (ρ= 1 and 1.27, respectively). To do this, we computed the average of the last 100 simulations and by compartment. Then, we calculated the percentage difference between our antibiotic-specific models compared with the two baseline scenarios (GN-ARB using low- and high-risk scenarios). Figure E5 shows the percentage difference between each of the antibiotic-specific GN-ARB models, compared to the base GN-ARB model (for ρ=1 and ρ=1.27).

The complete script is available on Python Jupyter notebook at <https://bit.ly/2ZpucKh> (antibiotic_bacteriaSpecModels).

New antibiotic-specific parameters and initial conditions based on Table E4 and E1, respectively

*Parameters (estimated on Table E4):*

$\beta$_h {quinolones}_ = 0.00006171

$\beta$_h {cephalosporins}_ = 0.006831

$\beta$_h {carbapenems}_ = 0.0001766

$\rho$_{quinolones}_= 1.57

$\rho$_{cephalosporins}_= 1.30

$\rho$_{carbapenems}_= 1.01

*Initial conditions (extracted from Table E1, descriptive statistics):*

Z_C{quinolones}_ = 39.49%*N

Z_C{cephalosporins}_ = 28.85%*N

Z_C{carbapenems}_ = 5.60%*N

Z_H{quinolones}_ = 46.45%*Hospital population

Z_H{cephalosporins}_ = 41.16%*Hospital population

Z_H{carbapenems}_ = 3.10%*Hospital population

**Figure E5 Percentage difference between new antibiotic-specific and base GN-ARB models.** We used each corresponding ρ parameter for quinolones, cephalosporins and carbapenems-specific models.

## New models for bacteria specific GN-ARB transmission dynamics

To better understand bacteria-specific variations in terms of transmission dynamics, we looked at the existing literature on PubMed^[[1]](#footnote-1)^ to understand the magnitude of transmission parameters used by previous studies for GN-ARB. We compiled the most recent estimates of these studies and adjusted our models to different transmission rates accounting for bacteria-specific transmissibility and according to the literature on GN-ARB and recent systematic literature reviews ^6,12-20^.

We varied the transmission parameter in hospitals (β_H_) using three specific bacteria family and strains: *Enterobacteriaceae*, *Klebsiella pneumoniae, and Acinetobacter baumanii*. We used the less conservative parameters from the literature to understand the highest impact that the community might experience. For Enterobacteriaceae, we used β_H_=0.029 (95%CI=0.016–0.049).^14^ Models studying *Escherichia coli* showed relatively small transmission values,^6,15^ so we employed a specific model for *Klebsiella pneumoniae* as an example and given it concentrated a relatively large number of articles (β_H_=0.0096, 95%CI= 0.0075, 0.012).^16^ Finally, we also surveyed transmission values for *Acinetobacter baumanii* ^17-20^ and used the largest estimates found (β_H_=0.078, 95%CI=0.072, 0.095).^17^

Consequently, we simulated the evolutions of the different compartment of our base GN-ARB model by estimating the mean, minimum and maximum compartment values using those values extracted from the literature. Finally, we compared our mean -bacterium specific- estimates of all compartments for the last 100-time points simulations against the base GN-ARB model (using ρ = 1 and 1.27). Then, we calculated the percentage difference between each compartment of the bacterium-specific mean estimate, compared to our base GN-ARB models. We computed the mean, minimum and maximum percentage values for the variation between models. Figure E6 shows the percentage difference between the exampled bacteria-specific GN-ARB models, compared to the base GN-ARB model (for ρ=1 and ρ=1.27).

The complete script is available on Python Jupyter notebook at <https://bit.ly/2ZpucKh> (antibiotic_bacteriaSpecModels).

**Figure E6 Percentage difference between new bacteria-specific and base GN-ARB models.** Black brackets indicate 95% CIs.

# Section F. Additional references

1 Ferreccio, C. *et al.* Study protocol for the Maule Cohort (MAUCO) of chronic diseases, Chile 2014–2024. *BMC public health* **16**, 122 (2015).

2 Araos, R. *et al.* in *Society for Healthcare Epidemiology of America (SHEA)* Vol. 6th (Atlanta, EEUU, 2020).

3 Bralic, R. A. *et al.* Colonization With Antibiotic-Resistant Gram-Negative Bacteria in Population-Based Hospital and Community Settings in Chile. *Infection Control & Hospital Epidemiology* **41**, s175-s176 (2020).

4 Cifuentes, M. *et al.* Grupo Colaborativo de Resistencia Bacteriana, Chile: recomendaciones 2014 para el control de la resistencia bacteriana. *Revista chilena de infectología* **32**, 305-318 (2015).

5 Allel, K. *et al.* Socioeconomic factors associated with antimicrobial resistance of Pseudomonas aeruginosa, Staphylococcus aureus, and Escherichia coli in Chilean hospitals (2008–2017). *Rev Panam Salud Publica; 44, jul. 2020* (2020).

6 Knight, G. M. *et al.* Quantifying where human acquisition of antibiotic resistance occurs: a mathematical modelling study. *BMC medicine* **16**, 137 (2018).

7 Allel, K. *et al.* Ch. 4, 115-151 (Centro de Políticas Públicas UC. *Propuestas para Chile. Concurso de Políticas Públicas 2019. Pontificia Universidad Católica*, 2020).

8 Heffernan, J. M., Smith, R. J. & Wahl, L. M. Perspectives on the basic reproductive ratio. *Journal of the Royal Society Interface* **2**, 281-293 (2005).

9 Van den Driessche, P. Reproduction numbers of infectious disease models. *Infectious Disease Modelling* **2**, 288-303 (2017).

10 Spellberg, B. *et al.* The epidemic of antibiotic-resistant infections: a call to action for the medical community from the Infectious Diseases Society of America. *Clinical infectious diseases* **46**, 155-164 (2008).

11 World Health Organization. *Antimicrobial resistance: global report on surveillance*. (World Health Organization, 2014).

12 Niewiadomska, A. M. *et al.* Population-level mathematical modeling of antimicrobial resistance: a systematic review. *BMC medicine* **17**, 1-20 (2019).

13 Knight, G. M. *et al.* Mathematical modelling for antibiotic resistance control policy: do we know enough? *BMC Infectious Diseases* **19**, 1-9 (2019).

14 Gurieva, T. *et al.* The transmissibility of antibiotic-resistant Enterobacteriaceae in intensive care units. *Clinical Infectious Diseases* **66**, 489-493 (2018).

15 Wickramasinghe, N. H. *et al.* High community faecal carriage rates of CTX-M ESBL-producing Escherichia coli in a specific population group in Birmingham, UK. *Journal of antimicrobial chemotherapy* **67**, 1108-1113 (2012).

16 Crellen, T. *et al.* Transmission dynamics and control of multidrug-resistant Klebsiella pneumoniae in neonates in a developing country. *Elife* **8**, e50468 (2019).

17 Wang, X. *et al.* A data-driven mathematical model of multi-drug resistant Acinetobacter baumannii transmission in an intensive care unit. *Scientific reports* **5**, 1-8 (2015).

18 Doan, T. N., Kong, D. C., Marshall, C., Kirkpatrick, C. M. & McBryde, E. S. Modeling the impact of interventions against Acinetobacter baumannii transmission in intensive care units. *Virulence* **7**, 141-152 (2016).

19 Doan, T. N., Kong, D. C., Marshall, C., Kirkpatrick, C. M. & McBryde, E. S. Characterising the transmission dynamics of Acinetobacter baumannii in intensive care units using hidden Markov models. *PLoS One* **10**, e0132037 (2015).

20 Talaminos, A. *et al.* Modelling the epidemiology of Escherichia coli ST131 and the impact of interventions on the community and healthcare centres. *Epidemiology & Infection* **144**, 1974-1982 (2016).

1. (("transmissability"[All Fields] OR "transmissable"[All Fields] OR "transmissibilities"[All Fields] OR "transmissibility"[All Fields] OR "transmissible"[All Fields] OR "transmissibles"[All Fields] OR "transmission"[MeSH Subheading] OR "transmission"[All Fields] OR "transmissions"[All Fields]) AND ("dynamer"[All Fields] OR "dynamers"[All Fields] OR "dynamic"[All Fields] OR "dynamical"[All Fields] OR "dynamically"[All Fields] OR "dynamicity"[All Fields] OR "dynamics"[All Fields] OR "dynamism"[All Fields] OR "dynamisms"[All Fields]) AND ("klebsiella pneumoniae"[MeSH Terms] OR ("klebsiella"[All Fields] AND "pneumoniae"[All Fields]) OR "klebsiella pneumoniae"[All Fields] OR ("escherichia coli"[MeSH Terms] OR ("escherichia"[All Fields] AND "coli"[All Fields]) OR "escherichia coli"[All Fields]) OR ("enterobacteriacea"[All Fields] OR "enterobacteriaceae"[MeSH Terms] OR "enterobacteriaceae"[All Fields]) OR ("acinetobacter baumannii"[MeSH Terms] OR ("acinetobacter"[All Fields] AND "baumannii"[All Fields]) OR "acinetobacter baumannii"[All Fields]) OR ("pseudomonas aeruginosa"[MeSH Terms] OR ("pseudomonas"[All Fields] AND "aeruginosa"[All Fields]) OR "pseudomonas aeruginosa"[All Fields]) OR "gram-negative"[All Fields])) AND (2001:2022[pdat]) [↑](#footnote-ref-1)
